# Toward the Digitalization of Sensory Analysis of Black Table Olives Using Electronic Nose, Tongue, and Eye

**DOI:** 10.3390/s26216826

**Published:** 2026-10-28

**Authors:** Ramiro Sánchez

**Affiliations:** Centro de Investigaciones Científicas y Tecnológicas de Extremadura (CICYTEX), Instituto Tecnológico Agroalimentario de Extremadura (INTAEX), Avenida Adolfo Suárez s/n, 06007 Badajoz, Spain; ramiro.sanchez@juntaex.es

**Keywords:** electronic nose, electronic tongue, electronic eye, table olives, sensory digitalization, PLS-DA, data fusion, CIELAB, farmyard-like off-odor, chemometrics

## Abstract

Trained sensory panels are the reference for evaluating odor, taste, and appearance, but routine use close to production is limited. This proof-of-concept study evaluated digital support for the sensory analysis of Californian-style black table olives using five electronic sensing blocks. The same 15 cans from three sensory-characterized batches were analyzed using the portable S3+ electronic nose and OECA electronic eye, together with the Integrated Electronic Sensory Analysis Platform (PIASE) at CICYTEX, which incorporates the ASTREE electronic tongue to provide the gustatory domain and, as reference systems, the HERACLES electronic nose and IRIS electronic eye. Can-level PLS-DA balanced accuracies were 1.000 for HERACLES, 0.933 for ASTREE, 0.867 for OECA, 0.867 for IRIS, and 0.533 for S3+. Because each condition was one production batch, these values measure discrimination among three batches, not validation of a general defect classifier. S3+ mean response followed the sensory ordering of off-odor intensity and OECA between-fruit color heterogeneity followed the panel ordering of color uniformity; both are descriptive three-batch concordances. OECA-calibrated color agreed with a CR-400 colorimeter within ΔE00 ≈ 1.2. Mid-level fusion did not outperform the best individual blocks. The results support complementary, domain-specific instruments to assist trained sensory panels rather than replace them.

## 1. Introduction

Sensory analysis is the reference approach for integrating odor, taste, texture, and appearance in foods, but trained panels are resource-intensive: assessors require selection and maintenance, sessions have limited throughput, and results are not continuously available for routine process control. In table olives, this limitation is especially relevant because commercial quality and defect assessment involve several sensory domains that cannot be captured by a single analytical signal [1,2,3,4]. The practical problem addressed by panel digitalization is therefore not to replace trained assessors with one classifier, but to obtain objective, repeatable, traceable, and potentially on-site instrumental measurements that can pre-screen samples, document deviations, reduce routine panel workload, and reserve human assessment for confirmation and regulatory or high-value decisions [2,3,5].

Electronic sensing technologies provide complementary access to this multidimensional space. Electronic noses capture volatile fingerprints and have been applied to table olive quality and off-odor discrimination [1,6,7,8]. Potentiometric electronic tongues measure cross-sensitive electrochemical fingerprints of aqueous olive pastes and brines; previous table olive studies have classified sensory defects and quantified acid, bitter, and salty intensities when calibration samples carried matched panel scores [2,3,5]. In Californian-style black olives, electronic tongue responses have also been related to thermal processing, phenolic content, and sensory defect intensity [9]. Conventional CIELAB colorimetry provides objective point measurements, whereas electronic eye and computer vision systems extend color assessment to spatial uniformity, morphology, and visible defects [10,11,12,13]. Multisensor fusion can integrate these domains, but improved classification is not guaranteed merely by increasing the number of sensing blocks [14,15,16,17].

The sensing platforms were selected using three criteria: prior evidence of application to table olives or closely related matrices, joint coverage of the three sensory domains evaluated by the panel (odor, taste, and appearance), and suitability for use close to production. Metal–oxide electronic noses have discriminated table olive quality and abnormal fermentations [1,6,7,8]; potentiometric electronic tongues have classified olive defects and quantified basic tastes from pastes and brines [2,3,5,9]; and CIELAB-based imaging has been applied to olive color and defect assessment [10,11,12,13]. The portable S3+ and the smartphone-based OECA were therefore chosen as the on-site core, ASTREE as the brine block for the gustatory domain, and HERACLES and IRIS, available in the PIASE shared facility, as commercial references against which the portable blocks could be benchmarked on the same samples.

A central methodological issue is that instrumental discrimination and sensory concordance are different questions. A model may recognize production batches because of any stable chemical or physical difference, while panel digitalization requires that the instrumental response can be linked to the same sensory domain evaluated by the assessors. This is particularly important when instruments interrogate different matrices—headspace, brine, crushed fruit, or whole-fruit images—and when chemical variables such as pH, salt, organic acids, or phenolic compounds can influence sensor fingerprints without being identical to perceived taste. In table olives, oleuropein and related secoiridoids are major contributors to bitterness, while processing changes oleuropein, hydroxytyrosol, tyrosol, and other phenolic derivatives [18,19,20]. A rigorous digitalization study must therefore preserve sample identity across platforms and interpret each sensing block against the appropriate sensory and physicochemical context.

The present work used the same 15 physical cans from three sensory-characterized production batches of Californian-style oxidized black table olives. The portable sensing core comprised the S3+ portable electronic nose and the OECA portable electronic eye; ASTREE added a direct brine gustatory block, and HERACLES and IRIS served as commercial olfactory and visual references. OECA color was independently checked against a Konica Minolta CR-400 colorimeter. Brine pH, chloride, titratable acidity, and a targeted phenolic profile were used to interpret the gustatory results. Maintaining Can_ID across platforms enabled true can-level comparison and mid-level data fusion rather than synthetic pairing of different units.

We hypothesized that the sensing domains would capture complementary, rather than interchangeable, components of the panel profile, and that classification performance would not necessarily parallel sensory concordance. The objectives were to: (i) characterize the sensory structure of conforming, mild, and strong farmyard-like off-odor batches; (ii) determine how olfactory, gustatory, and visual measurements relate to the corresponding panel domains and to relevant physicochemical/phenolic variables; (iii) compare can-level PCA and PLS-DA across the five platforms under identical cross-validation; and (iv) test mid-level fusion of S3+ + OECA, with and without ASTREE. Because one production batch represented each sensory condition, the study is a proof of concept for matched-can multisensory digitalization, not an externally validated defect classifier. The novelty of the study does not lie in the hypothesis that electronic sensing complements sensory analysis, which has been supported in other foods [14,15,16,17], but in its experimental test: the same 15 physical cans were measured by five platforms covering three sensory domains, evaluated with identical can-level folds and permutation tests, with the portable electronic eye calibrated against a color chart and checked against a reference colorimeter, and with discrimination and sensory concordance treated as separate questions.

## 2. Materials and Methods

### 2.1. Experimental Design and Samples

Three production batches of Californian-style oxidized black table olives, cv. Manzanilla Cacereña (as declared by the supplier), supplied by Olives & Pickles S.L., were investigated. Canned black ripe olives undergo thermal sterilization during commercial processing, providing microbiological stability and extended shelf life during storage [9,21,22]. The supplier had initially preselected the batches by its routine sensory quality control assessment as conforming (C), mild farmyard-like off-odor (DGS), or strong farmyard-like off-odor (DGF). This prior classification was used only to select contrasting material. The batches were subsequently and independently characterized by the trained CICYTEX sensory panel on 29 July 2026, whose results constitute the sensory reference used in this study. Five cans were selected from each batch, giving 15 matched physical cans. Because each sensory condition is represented by a single production batch, condition and batch are confounded by design; the study therefore evaluates discrimination among these three sensory-characterized batches rather than generalization to new production batches.

**Figure 1:**
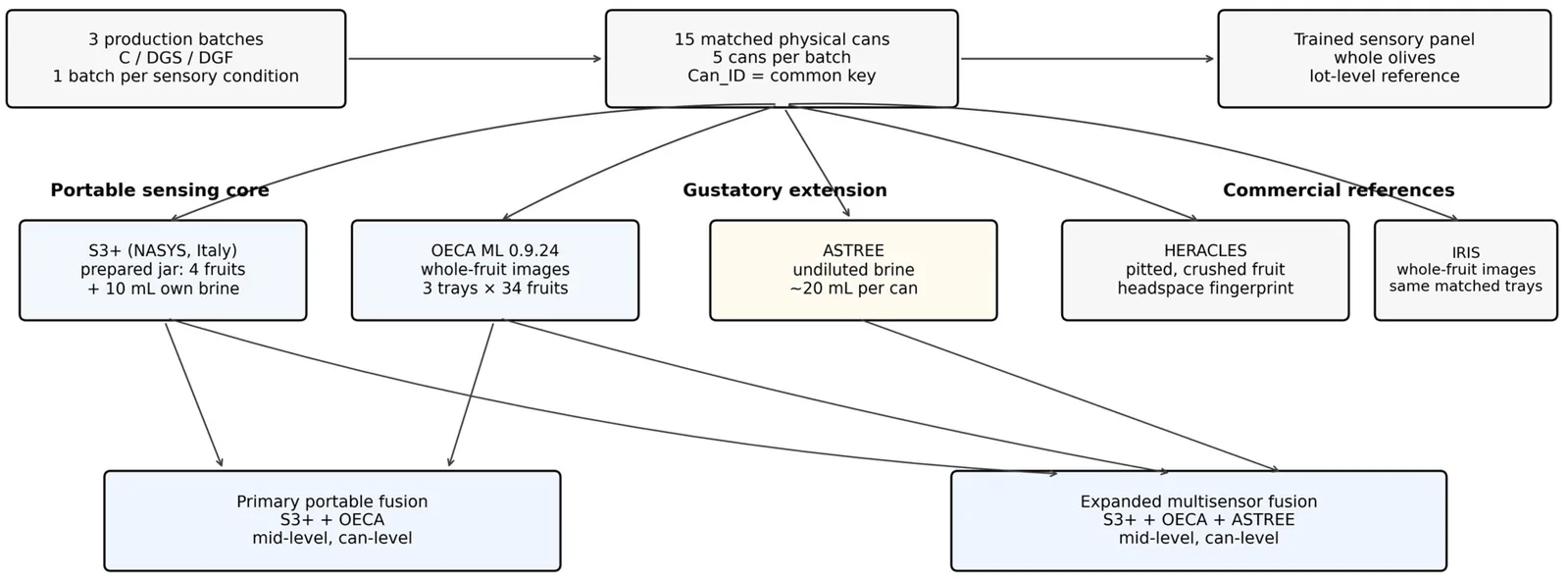
Matched-can experimental hierarchy and sensing roles. S3+ and OECA form the portable sensing core; ASTREE extends the brine/gustatory domain; HERACLES and IRIS are commercial references. Mid-level fusion used S3+ + OECA, with and without ASTREE.

The same physical cans were analyzed across all five sensing platforms, with each platform using the appropriate aliquot or subsample from the corresponding can. Can_ID (C_1–C_5, DGS_1–DGS_5, DGF_1–DGF_5) was the common join key. Because the platforms measured the same cans on different dates rather than simultaneously, the approach is referred to as matched-can, cross-platform fusion; measurement dates are given per block in Table 1 and per can in Table S1. Technical cycles, injections, sensor repetitions, trays, fruits, and pixels were never treated as independent cans. The experimental hierarchy and sensing platform roles are summarized in Table 1 and Figure 1.

Cans were opened in March 2026. Table S1 reports the recorded date of each measurement rather than assuming a single common opening date. Brine physicochemical measurements were recorded between 16 and 23 March 2026. HERACLES measurements were acquired on 20 March 2026 and ASTREE on 6 April 2026. After analytical sampling, the remaining olives were kept immersed in their own brine in hermetically closed containers at 5 °C until the sensory session (29 July 2026), S3+ measurements (15–18 September 2026), and OECA/IRIS imaging (30 September 2026).

### 2.2. Sensory Evaluation

A trained panel of nine assessors evaluated the three coded batches on 29 July 2026 using a continuous 1–11 intensity scale. For each batch, the panel sample was taken from additional, previously unopened cans of the same production batch, opened for the session, because material intended for human consumption must come from cans whose commercial sterility is intact; the 15 matched cans, already opened for the instrumental measurements, were therefore not used in the sensory session. Each assessor received four olives and 10 mL of their brine in a covered tasting glass at 20–25 °C, following International Olive Council recommendations, with the batches coded N1 (DGS), N2 (DGF), and N3 (C). The session followed an internal sheet based on the International Olive Council table olive sensory method [4], with an adapted visual block aligned with the Spanish table olive quality framework [23]. Marks on the 10 cm line were converted to the 1–11 scale. For the predominant perceived defect (DPP), the panel median (Me), interquartile range, robust standard deviation s* = 1.25 × IQR/(1.35 × √*N*), robust coefficient of variation (CVr), and 95% robust confidence interval were calculated following the adopted COI-based procedure. The farmyard-like descriptor was treated as an operational ‘other defect’ descriptor and was recorded by at least 50% of the panel. Results are from one sensory session and are used as sensory references rather than as a final official conformity decision. All assessors signed informed consent approved by the ethics committee before the session.

Critically, the panel evaluated the three production batches, not the 15 individual cans. The six panel variables were repeated in the master matrix only as lot membership metadata; they were not used as 15 independent sensory responses and no PLS regression against these repeated values was performed. The lot-level sensory references and their domain structure are presented in Section 3.1.

### 2.3. Portable Electronic Nose: S3+

**Figure 2:**
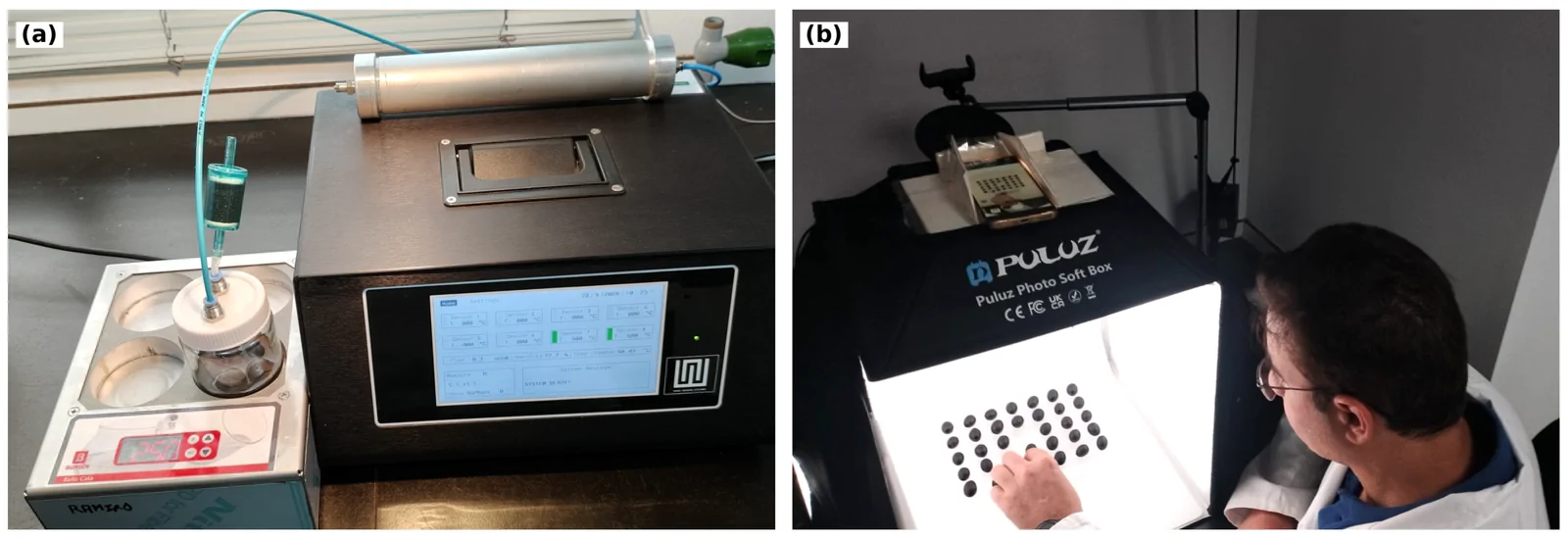
(**a**) S3+ portable electronic nose (Nano Sensor Systems, NASYS, Reggio Emilia, Italy) with the sample jar in the thermostatic bath; (**b**) OECA portable electronic eye: smartphone mounted on the LED light box (5500 K, CRI > 95) during an illustrative acquisition.

Measurements used the S3+ portable electronic nose (Nano Sensor Systems S.r.l. (NASYS), Italy), configured with two three-channel metal–oxide semiconductor sensor modules (six analytical channels: M1R1-M1R3 and M2R1-M2R3). For each can, four whole olives and 10.0 mL of their own brine were placed in a 190 mL vessel and conditioned for 5 min at 25 °C. Before the first measurement of each session, the instrument was stabilized for 60 min under activated carbon-filtered reference air. The 15 cans were measured in four sessions (15–18 September 2026) with the three batches interleaved within each session; acquisition time stamps and order are listed in Table S3. Chamber temperature and relative humidity were recorded by the instrument during each acquisition; ambient laboratory humidity was not controlled. Each acquisition comprised four cycles of 100 s activated carbon-filtered reference air, 45 s sample exposure, and 300 s filtered air cleaning/recovery at approximately 190–200 mL/min. Cycle 1 was treated as conditioning and excluded; cycles 2–4 were averaged (Figure 2).

For each channel *i*, the primary feature was the log-conductance ratio LGR*_i_* = ln(*G_i_*/*G*_0,*i*_) = ln(*R*_0,*i*_/*R*_f,*i*_), where *R*_0,*i*_ and *R*_f,*i*_ were the mean of the final five records in the reference air and sample exposure phases, respectively. The six LGR channels formed the S3+ classification block. The arithmetic mean of the six LGR channels was retained as a descriptive global intensity variable but was not added to the PLS-DA predictor block because it is a linear combination of the six LGR features.

### 2.4. HERACLES Commercial Olfactory Reference

Volatile fingerprints were obtained with a Heracles Neo flash gas chromatographic electronic nose (ref. HN-2211, Alpha MOS, Toulouse, France) equipped with a PAL-RSI autosampler and controlled with AlphaSoft 7.3.3. Two metal capillary columns of different polarity operated in parallel (MXT-5 and MXT-1701, 10 m × 0.18 mm i.d., 0.40 µm film), each coupled to a flame ionization detector, with hydrogen as carrier gas. For each can, 4 g of pitted, crushed olives without brine was placed in a 20 mL headspace vial and incubated at 70 °C for 15 min under agitation (500 rpm, 5 s on/2 s off). A 2000 µL headspace aliquot was injected into an injector at 200 °C; acquisition lasted 110 s. Three injections per can were averaged. Thirteen aligned peak area variables were exported, eight from the MXT-5 column and five from the MXT-1701 column. Kovats retention indices obtained with an n-alkane calibration mixture were used for peak alignment and tentative assignment in AlphaSoft/AroChemBase; compound assignments are treated as indicative only.

The 13 peaks were those selected in AlphaSoft by discrimination power among the three classes before export; because this selection used class information, the HERACLES classification result is optimistic and HERACLES is treated as a reference platform rather than as a validated classifier.

### 2.5. ASTREE Electronic Tongue

Taste-related fingerprints were obtained with an ASTREE electronic tongue (Alpha MOS, Toulouse, France) using sensor set #6, comprising seven cross-sensitive ChemFET (ISFET) sensors (AHS, PKS, CTS, NMS, CPS, ANS, and SCS) measured potentiometrically against a Ag/AgCl reference electrode with an LS48 autosampler, 25 mL beakers, and magnetic stirring. Before the session, the system completed the manufacturer’s conditioning–calibration–diagnostic sequence using 0.01 mol/L HCl, NaCl, and monosodium glutamate solutions. For each can, approximately 20 mL of undiluted brine at 20–25 °C was measured for 120 s at 1 Hz; the sensor value was the mean signal from 100 to 120 s. Three measurements per can were averaged, with distilled water rinsing between samples. Samples were interleaved by class (C, DGS, DGF), so class and run order were not confounded. Acquisition was performed on 6 April 2026. According to the manufacturer, sensor set #6 is intended to provide relative information related to acid, salty, and umami response domains; sensor labels are treated here as manufacturer designations rather than one-to-one taste measurements. ASTREE was used both as an individual block and as the gustatory extension of the portable S3+ + OECA system. Here, gustatory designates the sensory domain assigned to this block in the study design: ASTREE measures an electrochemical fingerprint of the brine, and no quantitative link to fruit taste scores was established in this study. Cross-sensitive potentiometric fingerprints should be interpreted multivariately rather than as one-sensor/one-taste measurements; because the array is based on ChemFET/ISFET technology, sensitivity to acid–base conditions is expected, but the response also reflects the broader ionic and molecular composition of the brine. Related applications in other food matrices also demonstrate the value of multivariate electronic tongue profiling [24,25]. In table olives, potentiometric electronic tongues have previously quantified acid, bitter, and salty intensities from olive pastes and brines and have classified organoleptic defects when matched sensory calibration data were available [2,3,5]; related work in Californian-style black olives showed sensitivity to thermal processing, phenolic, and sensory changes [9].

### 2.6. Brine Physicochemistry and Targeted Phenolic Profile

Brine pH, chloride, and titratable acidity were measured for each of the 15 cans in March 2026 using three replicate readings per determination; can-level means were used descriptively. pH was measured directly with a CRISON pH meter (Crison Instruments, Barcelona, Spain). Chloride concentration was determined by the Mohr argentometric method using 0.1 N AgNO_3_ and potassium chromate as an indicator and was expressed as % NaCl. Titratable acidity was determined by acid–base titration with 0.1 N NaOH using phenolphthalein as an endpoint indicator and was expressed as % lactic acid. These procedures follow commonly used table olive brine physicochemical methods [21,22]. These variables were not added to the PLS-DA predictor blocks; they were used to interpret the ASTREE direct brine fingerprint.

A targeted phenolic profile was determined directly in brine, the same matrix measured by ASTREE. Three analytical replicate preparations were performed per production batch sample. After homogenization, 10.00 mL of brine was extracted with ethyl acetate; the organic phases were combined, evaporated at low temperature, and the residue was reconstituted to 5.00 mL with MeOH:H_2_O 50:50 (*v*/*v*) and filtered through 0.22 µm before chromatographic analysis. Chromatography was performed on an Agilent 1290 Infinity III UHPLC system equipped with a diode array detector (DAD) (Agilent Technologies, Santa Clara, CA, USA), using an SB-C18 RRHD StableBond column (3.0 × 150 mm, 1.8 µm). Phenolic concentrations are reported as mg/L of original brine as reported by the laboratory worksheet and were used only as production batch chemical context, not as can-level classification or fusion variables [18,19,20].

### 2.7. Electronic Eye Acquisition

Each of the 15 cans provided three distinct fruit subsamples of 34 olives, giving 45 trays and 1530 fruit observations. The same fruit subsamples and tray arrangements were consecutively imaged by OECA and IRIS. The can remained the experimental unit; the three trays were subsamples used to estimate within-can repeatability and fruit-to-fruit heterogeneity.

Images were acquired with the main camera of a realme 12 Pro 5G smartphone (realme, Shenzhen, China; 26 mm equivalent focal length, f/1.8) in manual mode at ISO 100 and 1/100 s. Images were acquired inside a 40 × 40 cm foldable photo light box (Puluz Photo Soft Box, Puluz Technology, Shenzhen, China; 600D nylon, white diffusing walls) fitted with three LED panels totaling 480 LEDs (2 × 168 and 1 × 144), nominal color temperature 5500 K, color rendering index > 95, flicker-free, dimmable from 1 to 100%. The smartphone was fixed on the top mount of the box, facing down, at a constant camera-to-tray distance. The apparent correlated color temperature estimated from the gray patches of the ColorChecker was approximately 5500 K, consistent with the nominal value, with the camera white balance set manually to 5000 K. Images were stored as JPEG files with an embedded Display P3 ICC profile. No clipping at digital value 255 was observed in the fruit or chart regions used for analysis. A Calibrite ColorChecker Classic 24-patch chart (Calibrite LLC, Wilmington, DE, USA) was acquired at the beginning and end of the session [26], following established camera color calibration approaches using ColorChecker targets [27]. The initial chart image was used for color calibration, whereas the final chart image was used only to quantify within-session drift. An empty tray was also recorded at the beginning. Samples were acquired in class-wise blocks rather than in a fully randomized order; instrument stability during the imaging session was checked using the start/end ColorChecker images.

### 2.8. OECA ML 0.9.24 Portable Electronic Eye

Images were processed with OECA ML v0.9.24 (CICYTEX, 2026). The 45 tray images were processed using the same segmentation parameters throughout the campaign. Background was modeled by color within the automatically detected tray region (CHROMA_IN_ROI).

Color calibration used the native camera encoding. A per-channel tone curve was estimated from the six gray ColorChecker patches, followed by a 3 × 3 matrix to CIE XYZ (D50/2°) fitted on 23 patches; the cyan F3 patch was excluded because its reference color lay outside the reproducible camera gamut under the acquisition conditions. Reference values were the manufacturer-published D50/2° data for the ColorChecker generation introduced after November 2014; the physical chart used in this study was not individually certified. Leave-one-patch-out validation, with the held-out patch excluded from both the tone curve and the matrix, yielded a median ΔE00 of 4.53 (P95 7.25; median 3.68 for patches with L* < 40). Repeated chart photographs at the start and end of the session differed by a median ΔE00 of 0.51 (maximum 1.36). Color was expressed in calibrated CIELAB D50/2° rather than raw RGB because chart-referenced colorimetry permits cross-session and cross-instrument interpretation, and because between-fruit uniformity was quantified with CIEDE2000, a perceptual color difference metric [13].

The pre-specified reduced OECA predictor block comprised L*, a*, b*, P05 L*, the between-fruit SD of L*, and between-fruit ΔE00 median and P90. C* and h° were not added to the model because C* is derived from a* and b*, while hue is unstable at the low chroma (approximately 2–3) of these black olives. Additional morphology and texture descriptors were exported by OECA but were not included in the analyses reported here.

### 2.9. IRIS Reference and OECA-IRIS Comparison

The commercial IRIS VA400 visual analyzer (Alpha MOS, Toulouse, France) was used as the vision reference. The closed cabin (420 × 560 mm) provides independent top and bottom LED illumination (5700 K, CRI ≥ 70) and contains a Basler acA2500-14gc CMOS camera (Basler AG, Ahrensburg, Germany) (5 MP, 2590 × 1942 pixels, 12-bit) with a 5 mm lens, corresponding to an approximately 45 × 34 cm field and 5.8 pixels/mm. Acquisition and analysis were performed in AlphaSoft. The instrument was calibrated with its 24-patch color checker using the manufacturer wizard, which adjusts aperture and camera/acquisition parameters for repeatability; this procedure does not transform the camera output into chart-referenced CIELAB. Color is coded by the software on its 0–4096 scale and exported as RGB and L*a*b*. IRIS imaged the same 45 tray arrangements used by OECA. The principal cross-system comparison used L*, a*, and b* as relative within-system descriptors, while proprietary color bins and size classes were retained only for traceability. Absolute CIELAB coordinates were not treated as interchangeable because exposure settings, illumination geometry, and colorimetric reference differ between systems. Comparison therefore focused on standardized within-system values, rank association, between-batch structure, and repeatability.

### 2.10. Reference Colorimetry

On 2 October 2026, fruit from three cans (C_1, DGS_1, and DGF_5; 34, 35, and 32 fruits, respectively) was measured with a Konica Minolta CR-400 chroma meter (Konica Minolta Sensing, Osaka, Japan) using diffuse illumination/0° viewing geometry, specular component included, and an 8 mm aperture. The instrument was calibrated on its white plate before the session. Two readings were collected per fruit on opposite faces. Individual fruits were not matched between the two instruments; the comparison was therefore made between can-level mean L*a*b* coordinates (CR-400: mean of all readings of the can; OECA: mean of its three trays), and the ΔE00 values reported in Section 3.2 refer to these can means. CR-400 D65/2° coordinates were chromatically adapted to D50/2° using the Bradford transform to permit direct comparison with OECA. This reference check was not used in PCA, PLS-DA, fusion, or model selection. The same three cans were also imaged with IRIS using top illumination only on 2 October; these additional IRIS measurements were used only to examine the configuration dependence of absolute color coordinates.

### 2.11. Chemometric Analysis and Data Fusion

PCA was performed separately for S3+, OECA, ASTREE, HERACLES, and IRIS after mean centering and autoscaling. PCA was used descriptively to visualize unsupervised structure; no classification claim was based on PCA separation.

PLS-DA was evaluated for C, DGS, and DGF using PLS2 regression with three one-hot response columns (scikit-learn PLSRegression); the predicted class was the response column with the largest predicted value. Predictors were mean-centered and autoscaled using parameters fitted only on the relevant training data, and PLS internal scaling was disabled. The five outer folds were fixed by Can_ID: fold *k* left out C_*k*, DGS_*k*, and DGF_*k* and trained on the other 12 cans, so every can was predicted exactly once without entering its own training set. Identical outer folds were used for every platform. Within each outer training set, 1–3 latent variables were compared by an inner four-fold can-level validation using the remaining fold identifiers; balanced accuracy was the selection criterion and ties were resolved in favor of the smaller model. This nested structure follows standard PLS chemometric practice while reducing information leakage during preprocessing and model selection [28,29].

Model significance was assessed by 1000 can-label permutations (random seed 20261001). In each permutation, the complete C/DGS/DGF label attached to a physical can was shuffled, while all technical replicates remained nested within that can. The complete validation pipeline was rerun, including scaling, latent variable selection, PCA reduction for fusion, and outer fold prediction. The empirical permutation *p* value was calculated as (1 + number of permuted balanced accuracy values ≥ observed balanced accuracy)/(1000 + 1). This *p* value therefore tests whether the observed can-level discrimination exceeds that expected after breaking the link between instrumental data and batch labels; it is not a *p* value for an individual sensor variable.

Mid-level, matched-can cross-platform fusion was used rather than direct low-level concatenation. For each outer fold, S3+, OECA, and (for the expanded model) ASTREE were processed as separate blocks using only the 12 outer training cans. Each block was autoscaled and reduced independently by PCA to two scores, providing equal reduced dimensionality to olfactory, visual, and gustatory domains despite their different original variable counts. The fitted block-specific scalers and PCA loadings were then applied to the three held-out cans. Training scores were concatenated by the common Can_ID and autoscaled, after which the PLS-DA latent variable count (1–3) was selected by the inner can-level folds. Consequently, each physical can remained one observation: the primary fusion contained four PCA score predictor variables per can (two from S3+ and two from OECA), whereas the expanded fusion contained six (two from each of S3+, OECA, and ASTREE). The primary fusion combined S3+ + OECA; the expanded fusion added ASTREE. HERACLES and IRIS remained reference platforms outside fusion. No preprocessing, PCA loading, or PLS model was fitted using the held-out cans, preventing leakage from validation samples [14,15,29]. No PLS-R was fitted to panel scores because the panel supplied three lot-level reference profiles rather than 15 independent can-level responses.

Statistical and chemometric analyses were performed in Python 3.11.4; SciPy 1.17.1 and scikit-learn 1.9.0 were used for inferential statistics and multivariate modeling [30,31], while pandas 3.0.5/NumPy 2.4.6 were used for data handling and Matplotlib 3.11.1 for figures. The can-level predictor matrix and the analysis script that reproduces the can-level classification results of Section 3.6, Table S2, and the permutation tests are provided as Files S1 and S2. As a complementary check, three nonlinear classifiers with fixed hyperparameters (random forest with 500 trees, radial basis function SVM, and 3 nearest neighbors) were evaluated on the same outer folds without inner tuning, applied to the raw autoscaled variables of each block and, for the fused models, both to the within-fold PCA scores of the mid-level fusion and to the raw concatenated variables of all blocks (low-level fusion); PLS-DA with nested latent variable selection was repeated on the same inputs for reference (Table S4). Panel robust statistics followed the COI-based calculations described above. No inferential between-batch test was applied to sensory panel scores because the panel supplied only three batch-level sensory profiles. For brine physicochemical variables, can-level differences among the three batches were assessed by one-way ANOVA followed, when significant, by Tukey HSD pairwise comparisons; these tests are interpreted as comparisons among the three sampled production batches because each sensory condition corresponded to a single batch. Phenolic profiles were not subjected to inferential tests because the analytical replicates were replicate preparations of batch-level samples rather than independent cans.

## 3. Results

### 3.1. Sensory Structure of the Three Production Batches

**Figure 3:**
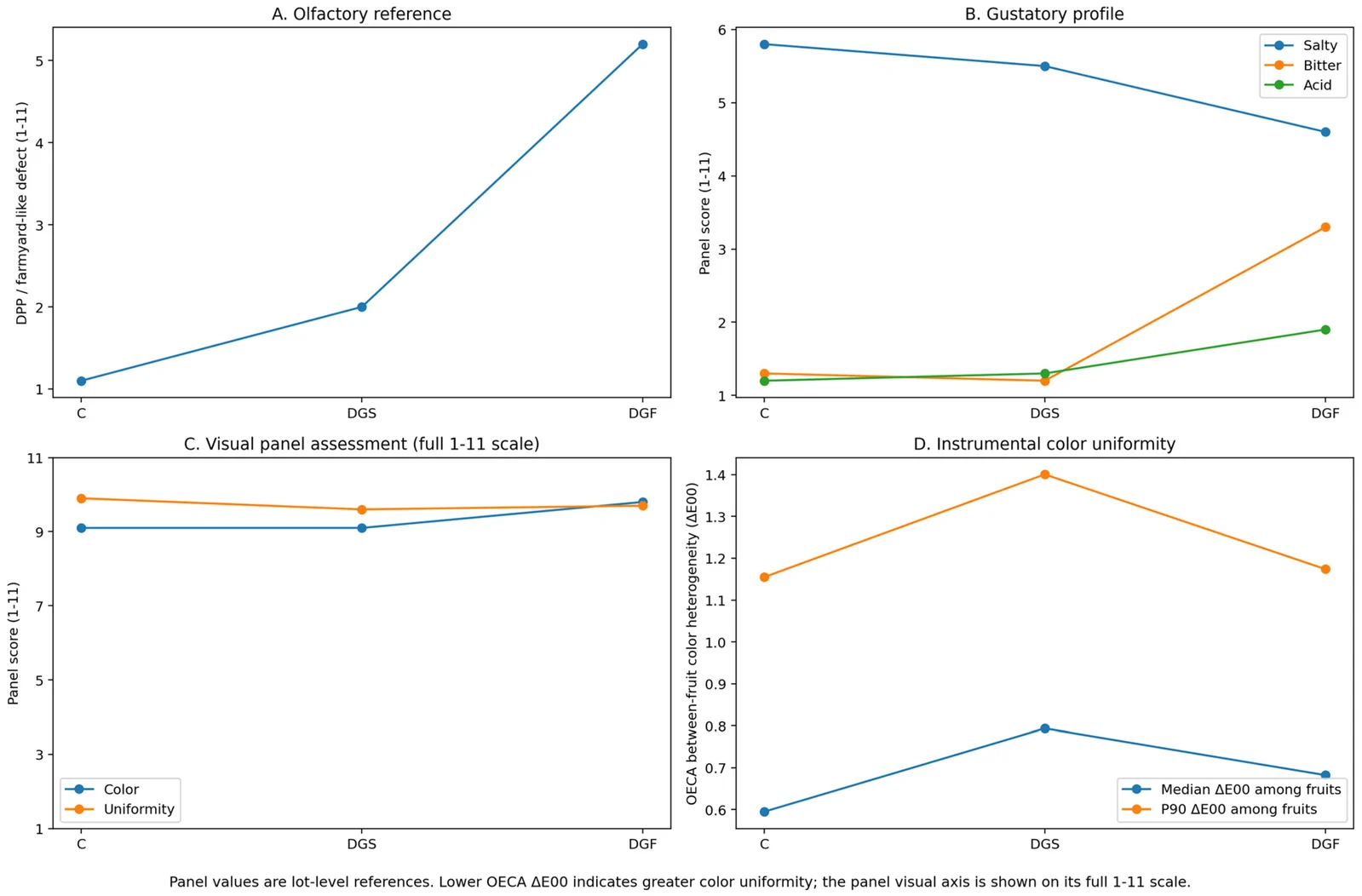
Sensory structure and visual concordance at production batch level. Panel values are lot-level references. OECA between-fruit ΔE00 is an instrumental uniformity measure; lower values indicate greater color homogeneity.

The panel established a clear olfactory gradient (Table 2). Median DPP/farmyard-like defect increased from 1.1 in C to 2.0 in DGS and 5.2 in DGF; robust CV values were 2.8%, 18.5%, and 19.6%, respectively. DGF also showed higher bitterness (3.3 versus 1.3 and 1.2 in C and DGS) and lower saltiness (4.6 versus 5.8 and 5.5). In contrast, all three batches received high visual scores (Figure 3). Thus, the strongest sensory separation was olfactory–gustatory rather than visual, while the visual domain provided a useful test of whether an electronic eye could objectively reproduce a narrow range of high color quality.

### 3.2. OECA Segmentation, Calibration, and Visual Descriptors

All 45 OECA tray images were segmented automatically with 34 detected olives per tray (1530 fruits in total), with no manual mask correction. The main calibrated color and uniformity descriptors by production batch are summarized in Table 3. Mean L* increased modestly from C to DGS/DGF, whereas the between-fruit color uniformity variables showed larger relative differences.

Between-fruit color heterogeneity followed the same batch ordering as the panel’s uniformity judgment. Median ΔE00 among fruits was 0.595 in C, 0.793 in DGS, and 0.682 in DGF; because lower ΔE00 indicates greater uniformity, the instrumental order C < DGF < DGS matched the panel order C > DGF > DGS. With three batches, any pre-specified strict ordering has a 1/6 probability of being matched by chance, and the within-batch ranges overlapped (median ΔE00 0.53–0.65 in C, 0.72–1.03 in DGS, and 0.45–0.86 in DGF); the agreement is therefore reported as a descriptive concordance, not as a validated can-level sensory relationship.

Reference colorimetry supported the absolute OECA color scale on the three checked cans: OECA–CR-400 ΔE00 ranged from 1.18 to 1.33, with |ΔL*| ≤ 0.97. The three-can comparison and between-fruit L* dispersion are summarized in Table 4. The ΔE00 values in Table 4 were computed from can-level mean coordinates. Their magnitude (1.2–1.3) is lower than the median leave-one-patch-out chart error (4.53) because the chart error is concentrated in saturated colors: in the leave-one-patch-out validation, the median ΔE00 was 6.0 for the ten patches with C* ≥ 40, 3.3 for the six neutral patches, and 2.7 for the two dark neutral patches (E4 and F4; L* 36 and 21). The olives (L* ≈ 24, C* ≈ 2–3) lie in this dark, low-chroma region, where the calibration error is smallest, so the colorimeter check is consistent with the chart validation. Because the calibration is one transformation applied to all fruits of a session, its residual error affects the absolute coordinates far more than between-fruit differences measured within the same image.

For the three CR-400-checked cans, IRIS L* was 31.4–32.2 under top illumination only on 2 October (mean ΔE00 to the colorimeter 5.8) and 41.0–41.8 under the standard matched-session configuration (mean ΔE00 13.7). IRIS absolute CIELAB outputs remain supplementary and are not treated as numerically interchangeable with OECA.

### 3.3. Relative Agreement Between OECA and IRIS

**Figure 4:**
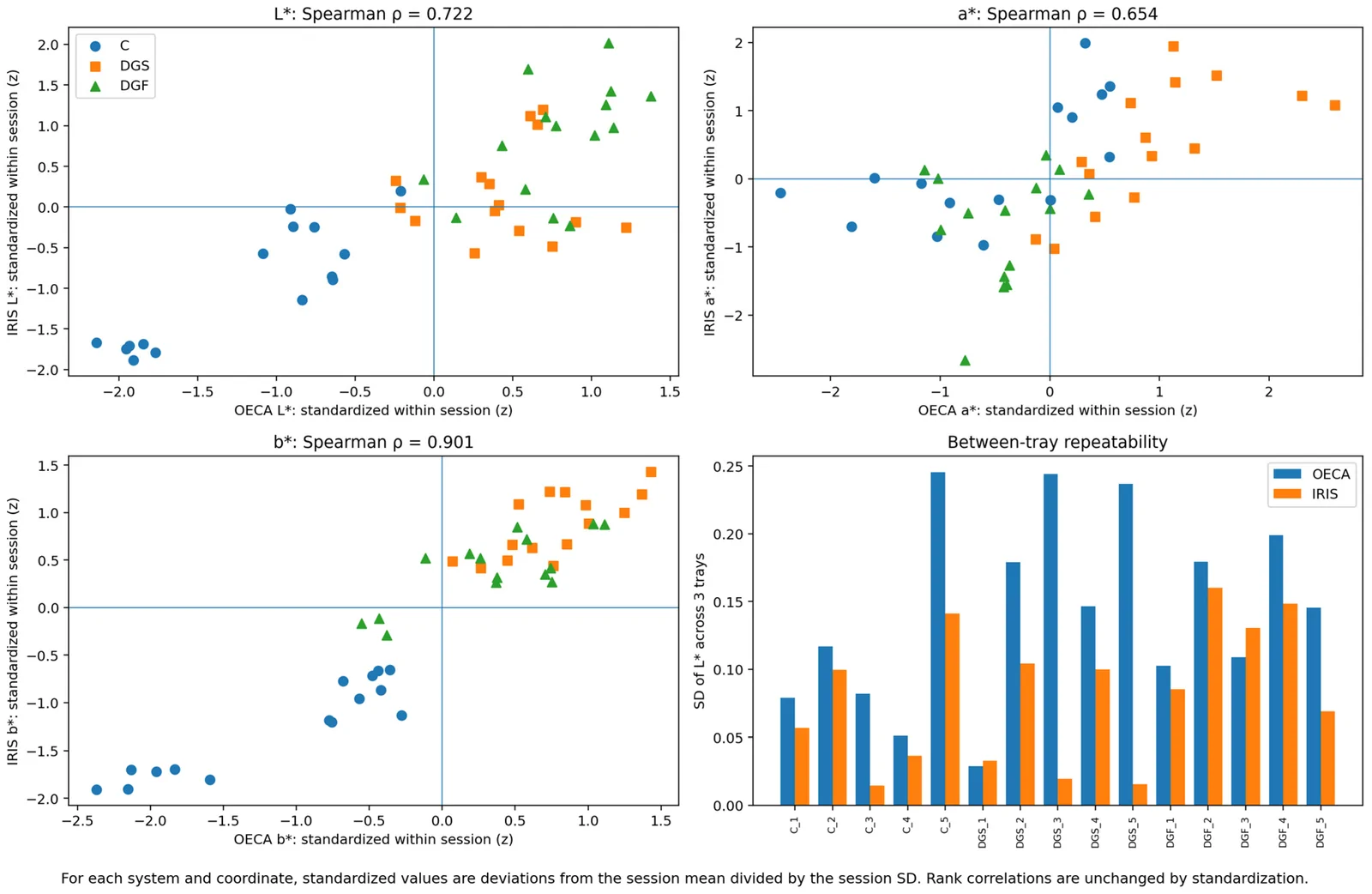
Relative color association and repeatability of OECA and IRIS on the same 45 trays. Each CIELAB coordinate was standardized within each system as the deviation from the session mean divided by the session SD before plotting. Spearman correlations are therefore unchanged. Absolute CIELAB coordinates were not treated as interchangeable.

On the same 45 trays, rank correlations between OECA and IRIS were *ρ* = 0.722 for L*, 0.654 for a*, and 0.901 for b*. After averaging the three trays within each can (*n* = 15), the corresponding correlations were 0.821, 0.643, and 0.914. Median between-tray SD of L* was 0.145 for OECA and 0.085 for IRIS. Figure 4 shows the standardized within-system comparison and repeatability. These results support comparison of relative within-system structure rather than absolute CIELAB equivalence.

### 3.4. Electronic Tongue Response and Gustatory Concordance

**Figure 5:**
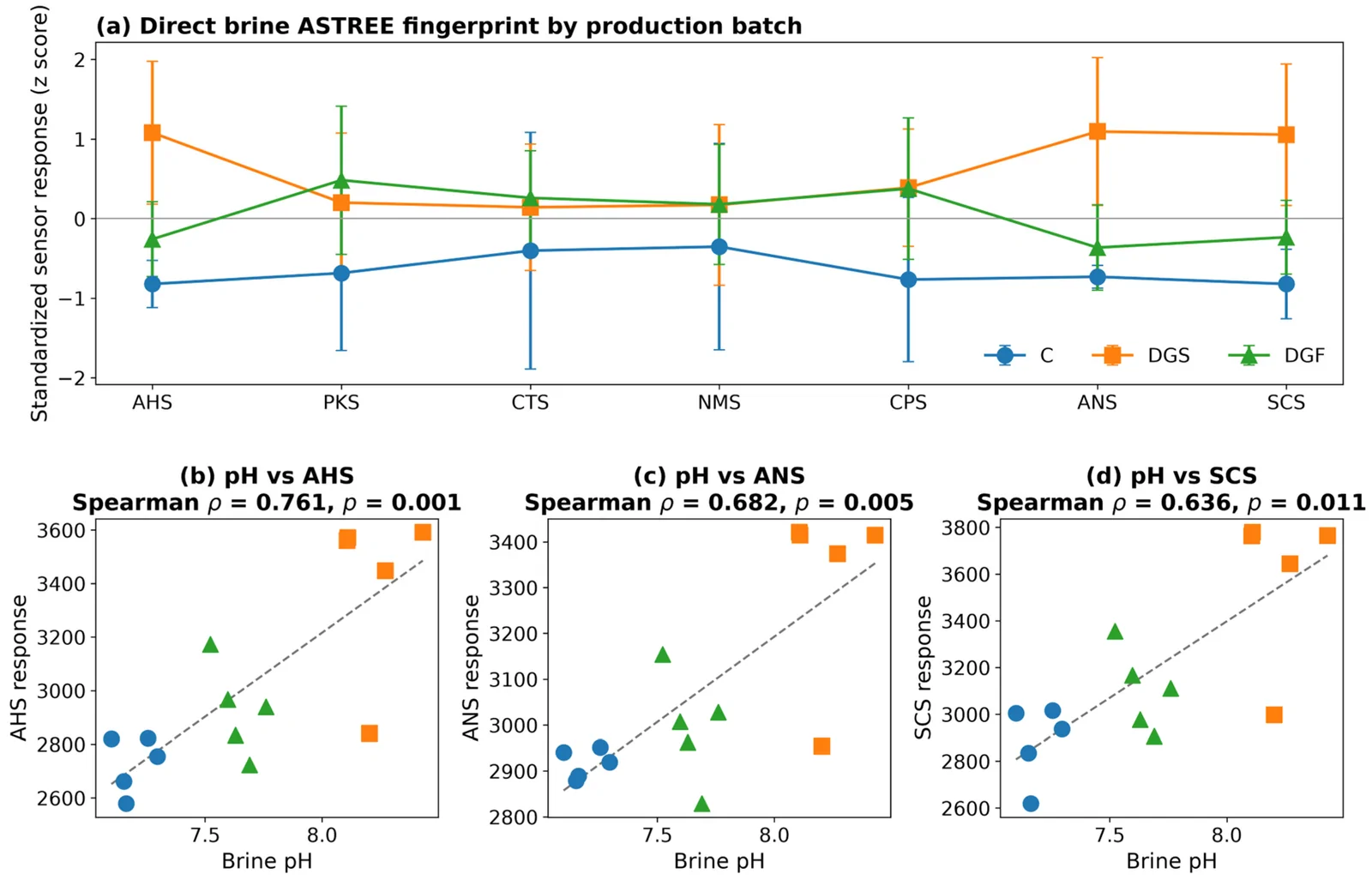
ASTREE direct brine responses. (**a**) Standardized seven-sensor profiles by production batch; error bars are SD across five cans. (**b**–**d**) Can-level brine pH versus AHS, ANS, and SCS. Spearman coefficients describe association, not causation; the ChemFET array remains cross-sensitive to the overall brine matrix.

ASTREE produced a strong direct brine fingerprint and classified 14/15 cans correctly, but this result should not be interpreted as direct prediction of fruit-level salty, bitter, or acid scores. The standardized seven-sensor profiles are shown in Figure 5 and batch means are reported in Table 5. AHS, ANS, and SCS showed the clearest batch shifts, with DGS giving the largest mean response in all three channels. Because the array contains potentiometric ChemFET/ISFET sensors, a strong contribution of acid–base and ionic conditions is expected; nevertheless, the channels are cross-sensitive and the fingerprint cannot be reduced to a pH measurement.

Can-level physicochemistry helped interpret this structure. Across the 15 cans, brine pH followed C < DGF < DGS and was rank-associated with AHS (Spearman *ρ* = 0.761), ANS (*ρ* = 0.682), and SCS (*ρ* = 0.636), whereas chloride showed no comparable association with those three channels. These correlations describe covariation among three batches that differ in pH rather than a can-level relationship: when ranks were computed within each batch and pooled, the association disappeared (*ρ* = −0.13, −0.27, and −0.23 for AHS, ANS, and SCS), so the nominal *p* values of the 15-can correlations (0.001–0.011) overstate the evidence, because the five cans of a batch are not independent realizations of the sensory condition. This pH covariance is mechanistically plausible for potentiometric ISFET/ChemFET sensing, but it does not establish pH as a unique causal driver because dissolved salts, organic acids, amino compounds, phenolics, and other ionic species contribute simultaneously. Published table olive studies likewise show that potentiometric electronic tongues require multivariate calibration against matched sensory or chemical references rather than one-sensor/one-taste assignments [2,3,5,9].

The complementary brine chemistry is summarized in Table 6. DGF had the lowest chloride concentration, matching the direction of its lower panel saltiness. More importantly, brine oleuropein increased in the order C < DGS < DGF, the same ordinal direction as panel bitterness (C ≈ DGS < DGF), whereas hydroxytyrosol was highest in C and tyrosol was highest in DGS. Mean PKS and CTS responses also increased C < DGS < DGF, but their within-batch dispersion was too large to assign a specific compound response. Thus, the ASTREE fingerprint appears to contain multiple chemical dimensions: some channels covary strongly with the acid–base state, while other parts of the multivariate response may track dissolved compounds with sensory relevance. No individual ASTREE channel is interpreted as a specific oleuropein, salt, or bitterness sensor without dedicated calibration. The quantified phenolic levels were of the same order of magnitude as those reported for packing brines of Californian-style black olives [19,20].

### 3.5. Unsupervised Instrumental Structure

**Figure 6:**
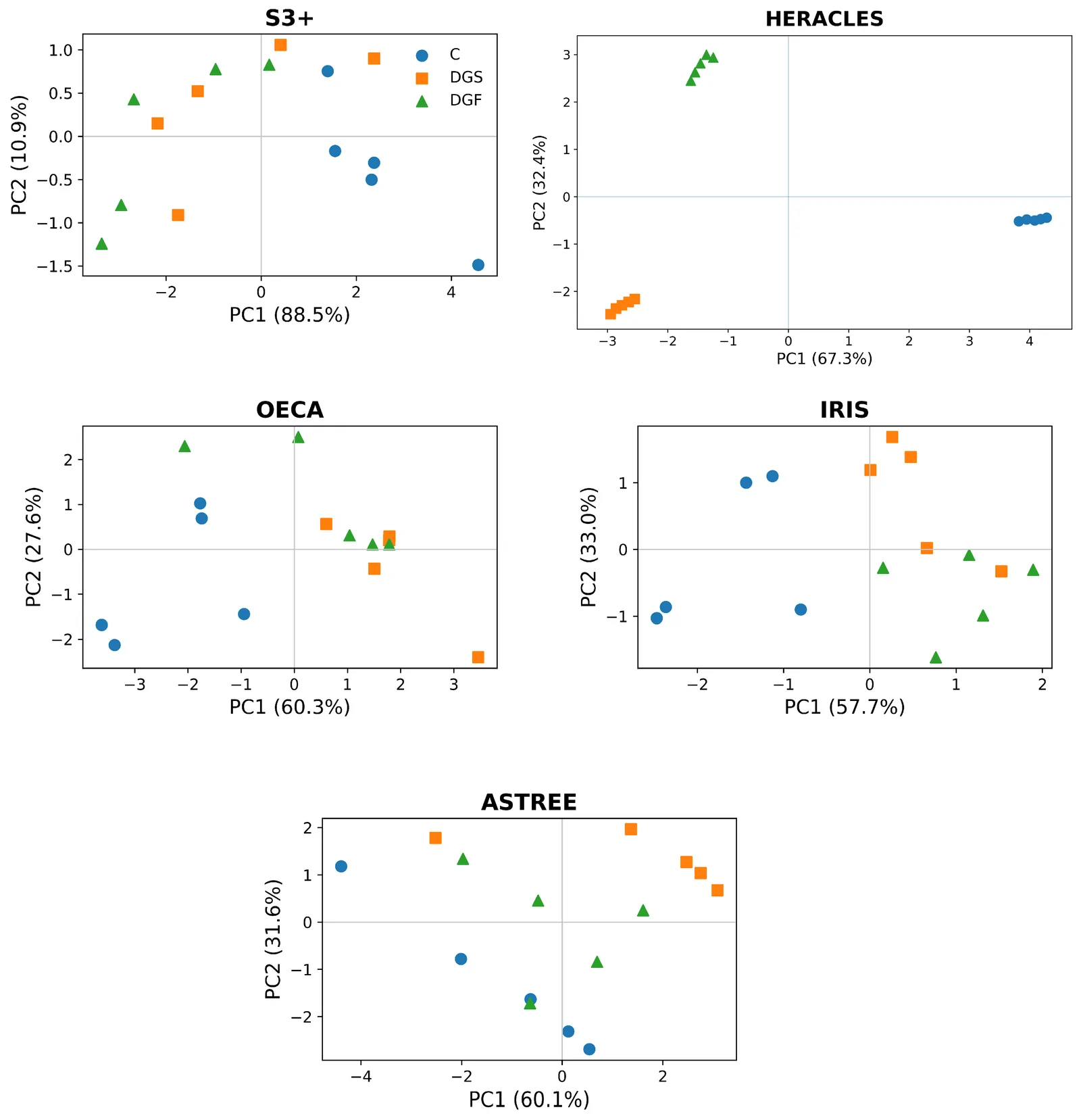
Unsupervised PCA of the five instrumental blocks, arranged by sensory domain (olfactory: S3+ and HERACLES; visual: OECA and IRIS; gustatory/brine: ASTREE). Each point is one physical can. HERACLES PCA uses the 13 AlphaSoft-selected peak area variables (8 MXT-5 and 5 MXT-1701) averaged across three injections per can; the other blocks use their pre-specified can-level variables. PCA was fitted after autoscaling and is used for visualization only.

PCA produced different variance structures across sensing domains. S3+ explained 88.5% and 10.9% in PC1 and PC2, respectively; its mean LGR intensity was 0.871 ± 0.049 for C, 0.745 ± 0.091 for DGS, and 0.676 ± 0.076 for DGF. Within-batch ranges of mean LGR overlapped: 0.833–0.954 in C, 0.666–0.882 in DGS, and 0.599–0.781 in DGF; three of the five DGS cans fell within the DGF range and two DGF cans within the DGS range (Kruskal–Wallis across batches *p* = 0.018; DGS versus DGF, Mann–Whitney *p* = 0.22). Recorded chamber temperature was 47–49 °C for 12 cans, whereas DGS_1 and DGF_4 were acquired in a higher regime (64–65 °C) and DGS_4 transitioned between regimes; chamber relative humidity ranged from 9 to 17% (Table S3). Excluding the two high-regime cans left the batch ordering of mean LGR unchanged (0.871, 0.765, and 0.683 for C, DGS, and DGF). HERACLES explained 67.3% and 32.4%, ASTREE 60.1% and 31.6%, OECA 60.3% and 27.6%, and IRIS 57.7% and 33.0% in the first two PCs. These PCA plots are descriptive only; class discrimination was assessed with out-of-fold PLS-DA rather than visual PCA separation (Figure 6).

### 3.6. Can-Level PLS-DA and Permutation Testing

**Figure 7:**
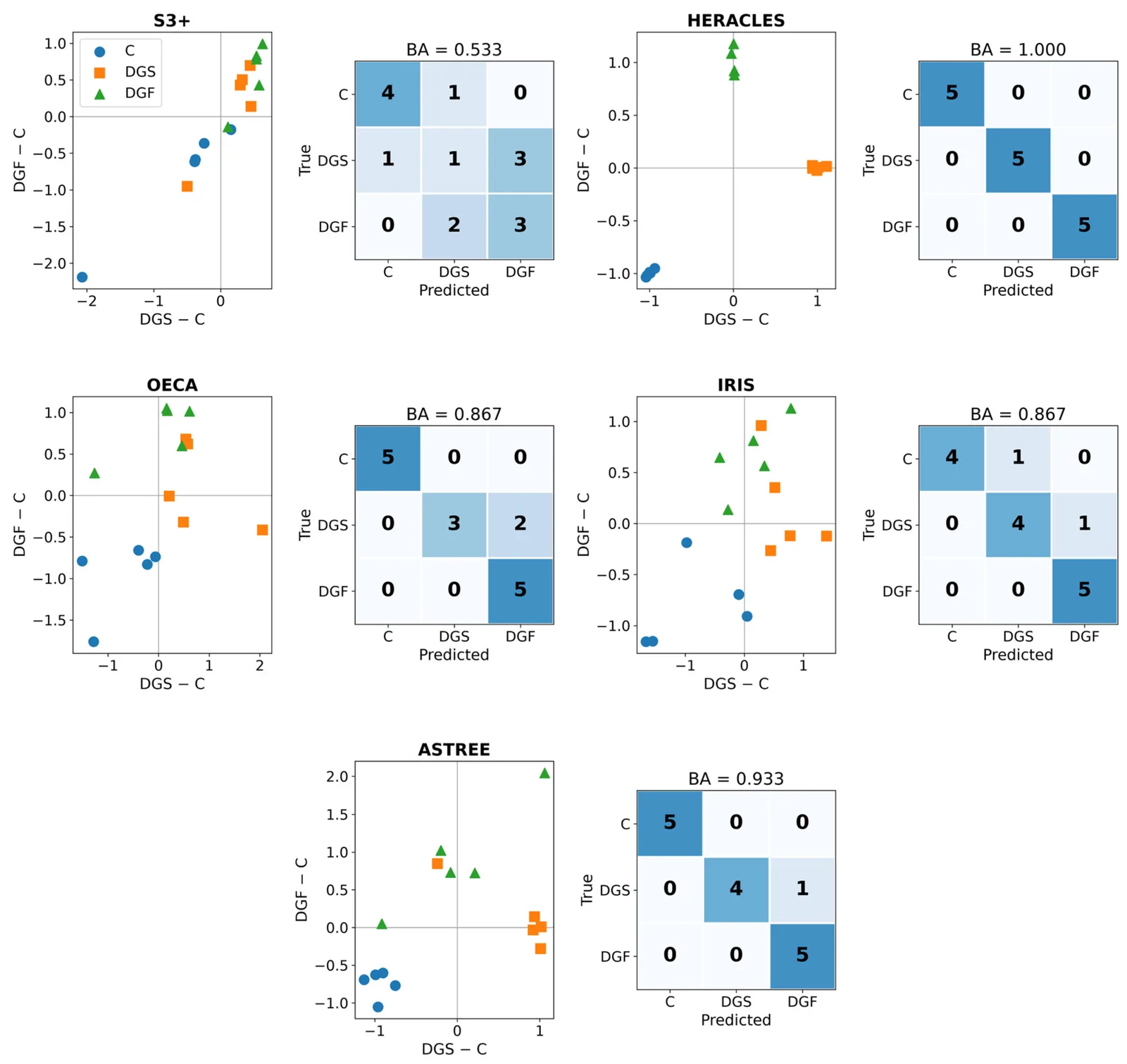
Out-of-fold PLS-DA class response coordinates and confusion matrices for the five instrumental blocks, arranged by sensory domain. Identical Can_ID folds were used across platforms; each fold left out one can from each batch. HERACLES uses the 13 AlphaSoft-selected peak area predictors averaged across three injections per can; because these predictors were selected by class-dependent discrimination power before export, outside the outer folds, the HERACLES result is optimistic and should be read as a reference platform result (Table 7 footnote). Confusion matrices use a light scale with black counts for legibility. Permutation *p* values from 1000 complete can label permutations are reported in Table 7.

Using identical can-level folds, HERACLES produced complete three-class separation using the 13 selected peak areas, followed by ASTREE, OECA/IRIS, and S3+. Figure 7 shows the out-of-fold class response coordinates and confusion matrices, while Table 7 reports balanced accuracy, macro precision, macro F1, overall accuracy, and can-level permutation *p* values. S3+ errors mainly reflected overlap between DGS and DGF despite its monotonic global response gradient. Because each condition is one production batch, these accuracies quantify discrimination among the three sampled batches rather than validated defect classification (Section 4, Limitations).

### 3.7. Mid-Level Fusion

**Figure 8:**
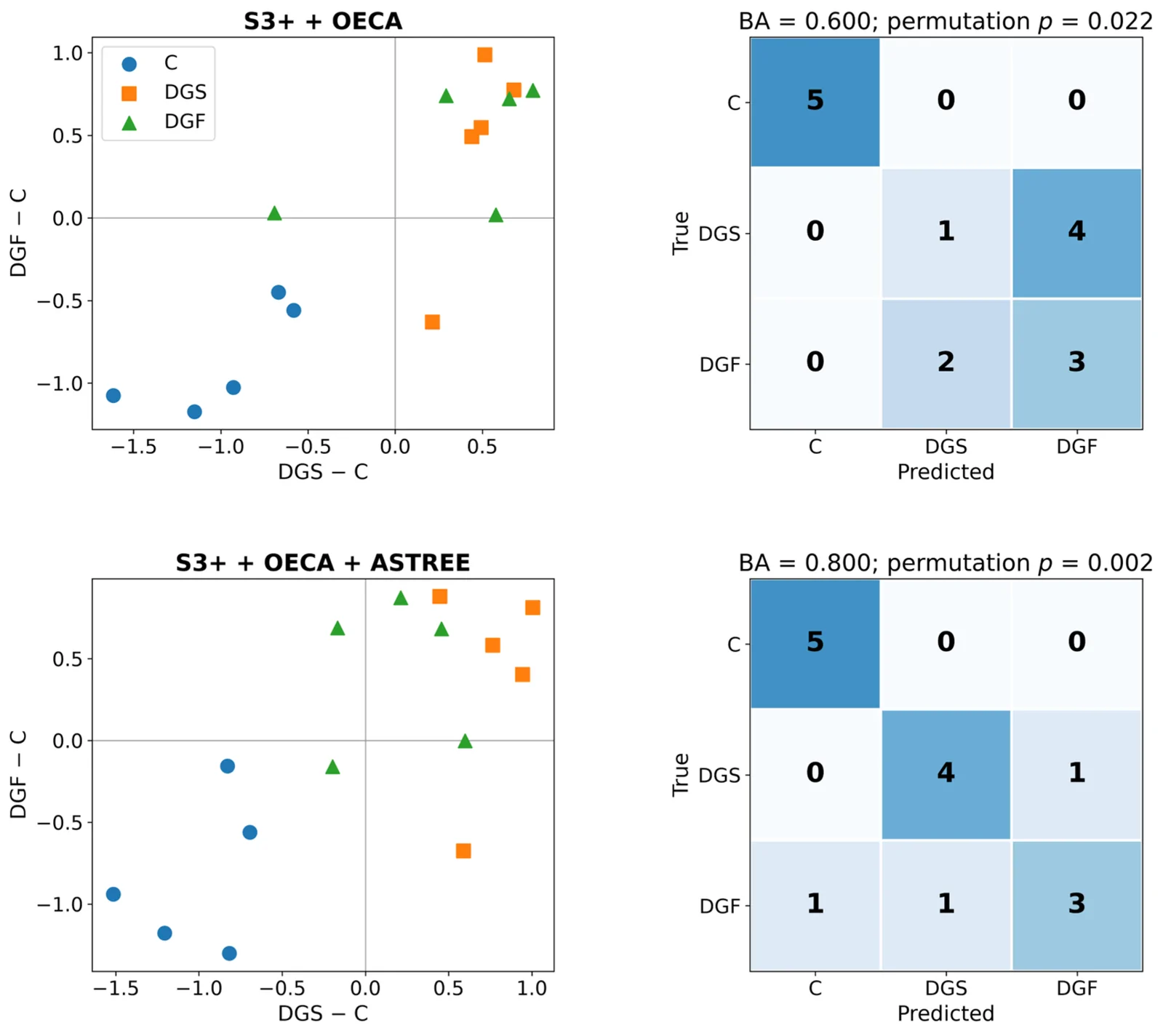
Mid-level fusion results. (Top) S3+ + OECA portable fusion and (bottom) S3+ + OECA + ASTREE expanded fusion. Each symbol is one physical can; the fused predictor vector contains four PCA scores per can for S3+ + OECA and six for S3+ + OECA + ASTREE. Out-of-fold confusion matrices and permutation *p* values are shown; the full permutation null distributions are provided as Figure S1.

The primary portable fusion S3+ + OECA did not improve on OECA alone. Adding ASTREE improved the expanded fusion, but performance remained below the strongest single blocks. With 15 cans from three batches, these results neither support nor refute a general benefit of fusion; they show only that, in this dataset, fusion did not outperform the best single block, and nonlinear classifiers evaluated on the same folds did not change this picture (Table S4). Figure 8 shows both fusion score spaces and confusion matrices. The corresponding 1000-permutation *p* values are reported in Table 7, and full permutation distributions are provided in Figure S1.

For the present proof of concept, the value of the portable core is functional rather than purely classificatory: S3+ measures the olfactory/headspace dimension, while OECA objectively quantifies the visual block that the nose cannot access. ASTREE adds a gustatory dimension, but the small batch-confounded dataset is insufficient to optimize the fusion architecture without high overfitting risk. The relationship between instrumental domains and panel assessment is summarized in Table 8, and Table 9 summarizes all chemometric models.

## 4. Discussion

The results support the working hypothesis that discrimination and sensory concordance are distinct dimensions of sensor performance (Table 8). This distinction is important for panel digitalization. Electronic sensing studies often emphasize classification accuracy, yet the practical objective in sensory support is to relate instrumental domains to perceptual attributes. Prior table olive electronic tongue studies achieved quantitative prediction of acid, bitter, salty, and negative defect intensities when many samples carried matched sensory scores and the olive/brine matrix was part of the calibration [2,3,5]. The present design instead asks a more preliminary question: whether matched instrumental domains preserve the sensory structure of three characterized batches without inventing can-level panel scores.

The visual results illustrate a comparatively direct domain link. CIELAB is widely used for objective olive color assessment, while computer vision adds spatial information unavailable from a point colorimeter [10,11,12,13]. OECA between-fruit ΔE00 matched the panel ordering of color uniformity, a descriptive three-batch concordance, and the CR-400 check supported its calibrated absolute color on three cans. This agreement is more meaningful for digitalization than simply separating C/DGS/DGF, because color heterogeneity corresponds to the same visual concept judged by the panel. IRIS showed similar relative ordering but, owing to different acquisition/color reference conditions, its absolute CIELAB values were not treated as interchangeable with OECA. The purpose of the OECA–IRIS comparison was not to show that two vision systems correlate, but to verify on identical trays that a smartphone-based portable eye recovers the same relative color structure and repeatability as a commercial reference, which is a prerequisite for using the portable device in panel support; the comparison also showed that the absolute CIELAB output of the commercial system depends on its illumination configuration, which argues for chart-referenced calibration in any digitalization workflow.

ASTREE provides a complementary and scientifically informative counterexample. Its high three-batch discrimination did not reproduce a simple ordinal scale of salty, bitter, and acid fruit scores because the instrument measured undiluted brine whereas panelists evaluated the fruit. As expected for an ISFET/ChemFET potentiometric array, part of the fingerprint covaried with brine pH, particularly AHS, ANS, and SCS; however, these sensors remain cross-sensitive to the complete ionic and molecular environment. Published table olive studies have quantified basic tastes when electronic tongue fingerprints were calibrated against sufficiently numerous matched sensory samples and have shown sensitivity to physicochemical and phenolic changes in Californian-style black olives [2,3,5,9]. The present result should therefore be read as a chemically informative brine fingerprint, not as evidence that ASTREE measures pH instead of taste.

The brine phenolic results add a second dimension to this interpretation. Oleuropein increased C < DGS < DGF, matching the ordinal direction of the panel bitterness medians, whereas the pH-associated AHS/ANS/SCS channels reached their maximum in DGS. This divergence supports a multicomponent interpretation of the tongue response: different regions of the sensor space can reflect different aspects of brine chemistry. Oleuropein and related secoiridoids are established contributors to olive bitterness, but alkaline oxidation and storage substantially transform the phenolic pool [18,19,20]. Hydroxytyrosol and tyrosol, which were abundant in the present brines, are mainly hydrolysis products and are not sufficient proxies for bitterness. Because phenolics were measured at production batch level rather than independently in all 15 cans, the apparent oleuropein–bitterness ordering is treated as descriptive concordance rather than inferential correlation.

Fusion likewise should not be judged only by whether concatenation increases classification accuracy. The primary portable fusion did not improve on OECA alone, and the expanded fusion remained below the strongest individual block while showing one-can sensitivity to the PCA nesting implementation. This argues against post hoc optimization aimed solely at maximizing accuracy and agrees with reviews emphasizing that data fusion benefit depends on block complementarity, preprocessing, and validation design [14,15]. The portable system remains valuable because it covers complementary olfactory and visual domains, not because fusion is guaranteed to outperform every single sensor block. In the present dataset, the fusion results are inconclusive rather than negative: 15 cans from three batches cannot establish or exclude a fusion benefit, and neither nonlinear classifiers applied to the raw block variables nor low-level fusion of the raw concatenated variables, linear or nonlinear, altered the picture on the same folds (Table S4): low-level fusion of the raw variables reached BA 0.733 for S3+ + OECA with every learner and at most 0.867 for S3+ + OECA + ASTREE (PLS-DA, random forest and kNN; 0.800 with SVM), i.e., equal to OECA alone and below ASTREE alone (0.933). Because PCA scores are mutually orthogonal and discard within-block structure, nonlinear learners were also applied to the raw variables rather than only to the fused scores. A dedicated comparison of fusion levels (low, mid, and decision level) on a multi-batch design remains to be done. Fusion was nevertheless pre-specified and is reported, because omitting unfavorable results would bias the literature on multisensor systems.

For olfaction, the contrast between S3+ and HERACLES also reflects the sensing principle. MOS electronic noses provide compact, portable cross-sensitive fingerprints and have previously discriminated table olive quality and abnormal fermentations [1,6,7], whereas flash GC electronic noses resolve chromatographic peak patterns and have shown strong discrimination in olive oil and other food applications [32,33,34]. HERACLES achieved complete out-of-fold separation of the three production batches using the 13 AlphaSoft-selected peak areas. Because those peaks were selected by discrimination power using the same class labels before export, the BA = 1.000 result is optimistic and HERACLES is treated as a reference platform rather than as a validated classifier. The strong separation indicates marked differences in the volatile fingerprints of these three batches, but it should be interpreted as batch discrimination rather than external validation of a general classifier for the farmyard-like defect. In contrast, S3+ showed lower three-class classification performance but displayed a monotonic global response gradient with increasing DPP severity. This behavior is consistent with the broader use of portable MOS electronic noses for table olive quality and spoilage screening [1,6,7].

A further strength is the matched-can design. All five platforms were linked through the same Can_ID, avoiding synthetic pairing of different cans from the same lot. This permits legitimate can-level data fusion. At the same time, matching cannot solve the more fundamental design limitation: only one production batch represented each sensory condition. Therefore, high performance indicates that cans from these three batches are distinguishable, not that a universal farmyard defect classifier has been externally validated. Several unmeasured batch properties could contribute to separation.

Visual acquisition order created an additional confounding between class and time because C, DGS, and DGF were photographed in blocks rather than alternated. The start/end chart comparison showed limited OECA color drift (median ΔE00 0.51; maximum 1.36), but this control cannot prove that every class difference is independent of time. More broadly, the same physical cans were measured across a six-month period: March physicochemistry/HERACLES, April ASTREE, July sensory analysis, and September S3+ and OECA/IRIS. Remaining olives were stored in their own brine in hermetically closed containers at 5 °C between measurements. Matching Can_ID therefore preserves sample identity but does not eliminate storage time effects. For the S3+ gradient, storage could have acted in either direction: loss of volatiles and slow oxidation at 5 °C would attenuate the total MOS response of all batches, whereas continued formation of farmyard-like compounds in the defective batches would increase their headspace load. HERACLES, acquired within days of opening, already separated the three batches, which indicates that the batch differences in volatile composition predate storage; however, the sign and magnitude of the S3+ mean response gradient at the time of the panel session cannot be reconstructed from the present data. The sensory reference itself was obtained from freshly opened cans of the same batches and is not affected by this storage. Future campaigns should measure all modalities within a much narrower time window, randomize or alternate class order, and include multiple independent production batches per sensory condition.

The next stage toward true panel digitalization should use a calibration set in which each independent sample or lot has its own sensory score for DPP, basic tastes, color adequacy, and color uniformity. With a sufficiently wide sensory range and multiple production batches per condition, PLS-R or other regression methods could then be validated in units of the 1–11 panel scale. The present study establishes the measurement architecture and the domain-specific links needed for that next step.

### Limitations

The study has five principal limitations. First, condition and production batch are confounded because there is one batch per C/DGS/DGF condition; high accuracy therefore means recognition of these batches, not generalization to new batches. Second, the sensory reference is at lot level and was obtained in one panel session from freshly opened cans of the same batches, not from the 15 matched cans; C and DGS were both provisionally classified as extra and only DGF as second/standard, so the experimental C/DGS/DGF labels are sensory-characterized batches rather than three official commercial categories. Third, ordinal sensory concordance is based on only three batch points: for any pre-specified strict ordering there are six possible permutations, so an observed ordering match has a nominal 1/6 chance under random ordering and must be treated as descriptive. Fourth, the same cans were characterized at different times over approximately six months while stored in their own brine at 5 °C, so storage time is confounded with platform; the visual campaign itself was also class-blocked in time, and two S3+ acquisitions (DGS_1 and DGF_4) were recorded in a higher chamber temperature regime. Fifth, the dataset is too small to optimize the fusion architecture without risking unstable model selection. External validation will require multiple independent batches per sensory condition, preferably from different production dates or producers, analyzed across the complete sensor suite with matched sample identifiers and harmonized acquisition timing.

## 5. Conclusions

The problem addressed by sensory panel digitalization is not the absence of a reference sensory method, but the difficulty of deploying trained panels continuously for rapid, traceable, and repeatable decisions close to production. A digital support layer should therefore capture complementary olfactory, gustatory, and visual information objectively, flag deviations, and preserve interpretable links to the corresponding sensory domains while leaving the trained panel as the calibration and adjudication reference.

The present matched-can study shows why discrimination and sensory concordance must be evaluated separately. A platform can recognize production batches very accurately without necessarily reproducing the ordering of a specific sensory attribute. Conversely, a simpler portable response may be valuable when it follows the relevant sensory direction. The combined architecture of portable olfaction and vision, extended by direct brine electronic tongue measurements and benchmarked against commercial reference systems, provides a technically coherent foundation for instrument-assisted panel support rather than an attempted one-device replacement of sensory analysis.

Accordingly, this work is a proof of concept for domain-specific digital support of sensory analysis, and its results suggest a concrete architecture for the next stage. (i) For on-site screening, the portable core should combine the S3+ electronic nose, used as a headspace fingerprint and intensity screen with a water blank and a fixed chemical reference, and the OECA electronic eye, used as a chart-referenced colorimetric and uniformity measurement. Of the two, chart-referenced color and between-fruit uniformity can be deployed now for objective documentation, because they are traceable to a physical reference and were consistent with a colorimeter; the electronic nose requires a multi-batch calibration before its gradient can be used as a screening criterion. (ii) Brine electrochemistry (ASTREE) and flash GC olfaction (HERACLES) belong in a laboratory tier as confirmatory and calibration instruments, HERACLES also serving to identify the volatile markers that a portable array should target. (iii) The data layer should keep the physical sample as the unit of identity across platforms, use nested can-level validation with permutation tests, and compare low-level, mid-level, and decision-level fusion only once a multi-batch calibration set exists, because the present data cannot rank them. (iv) In routine use, a trained panel can rely on such a system to pre-screen lots, document deviations, and select the samples that require sensory confirmation, while remaining the calibration and adjudication reference. Data fusion and validation approaches developed for olive oil and beverages [14,15,16,17,24,25] transfer directly to table olives at the modeling level, but the sensory calibration must be matrix-specific. The next decisive experiment is a multi-batch calibration study in which each independent sample receives its own panel scores and all instrumental domains are measured within a harmonized time window. That design will allow externally validated regression of DPP, basic tastes, color adequacy, and color uniformity in the panel scale, define operational uncertainty, and determine where digital sensing can reliably screen routine production and where trained sensory confirmation remains indispensable.

## Figures and Tables

**Table 1:** Experimental hierarchy and role of each data block. Abbreviations: DPP, predominantly perceived defect; LGR, logarithmic gas response; CIELAB L*, a*, b*, lightness, green-red, and blue-yellow color coordinates; OECA, portable electronic eye system; ML, machine learning. Dates are those of the measurements used in the analysis.

Block	Independent Unit Used in Models	Technical/Nested Observations	Primary Variables/Role	Measurement Date (s)
Sensory panel	Production batch (3 total)	9 assessors; single session	DPP/farmyard-like defect; salty, bitter, acid; color adequacy; color uniformity	29 July 2026 (one session)
S3+ (Nano Sensor Systems S.r.l. (NASYS), Brescia, Italy)Nano Sensor Systems S.r.l. (NASYS), Brescia, Italy)	Can (15), measured via a prepared jar from the corresponding can	Prepared jar: 4 whole fruits + 10 mL own brine; 4 cycles; cycle 1 excluded, cycles 2–4 averaged	Six logarithmic gas response channels [LGR = ln(*G*/*G*_0_)]; portable olfactory block	15–18 September 2026 (four sessions)
HERACLES	Can (15)	4 g pitted crushed fruit, no brine; 3 headspace injections averaged	13 aligned peak areas (8 MXT-5 and 5 MXT-1701; Kovats-indexed); commercial olfactory reference	20 March 2026
ASTREE	Can (15)	~20 mL undiluted brine; 3 measurements averaged	7 cross-sensitive potentiometric sensor channels; direct brine gustatory fingerprint	6 April 2026
OECA ML 0.9.24	Can (15)	3 trays × 34 whole fruits; fruit/pixel observations nested	CIELAB L*, a*, b* color coordinates and between-fruit CIEDE2000 uniformity; portable visual block	30 September 2026
IRIS	Can (15)	Same 3 whole-fruit trays used for OECA	CIELAB L*, a*, b* and proprietary color/size outputs; commercial vision reference	30 September 2026

**Table 2:** Sensory reference profile and session statistics used to interpret instrumental data. Gustatory and visual values are medians; *n* indicates the number of assessors who marked that line. DPP statistics derive from the nine-assessor session.

Sensory Attribute/Statistic	C	DGS	DGF
DPP/farmyard-like defect, median	1.1	2.0	5.2
DPP frequency	7/9	9/9	9/9
DPP CVr (%)	2.8	18.5	19.6
DPP robust 95% CI	1.0–1.2	1.3–2.7	3.2–7.2
Provisional single-session COI category	Extra	Extra	Second/standard
Salty, median (*n* marked)	5.8 (8)	5.5 (7)	4.6 (6)
Bitter, median (*n* marked)	1.3 (7)	1.2 (6)	3.3 (8)
Acid, median (*n* marked)	1.2 (6)	1.3 (7)	1.9 (8)
Color adequacy, median (*n* marked)	9.1 (7)	9.1 (6)	9.8 (7)
Color uniformity, median (*n* marked)	9.9 (8)	9.6 (8)	9.7 (5)

**Table 3:** Main visual descriptors by production batch (mean ± SD across five cans).

Descriptor	C	DGS	DGF
OECA L*	23.82 ± 0.48	25.00 ± 0.27	25.25 ± 0.27
OECA a*	0.568 ± 0.086	0.702 ± 0.064	0.577 ± 0.040
OECA b*	2.097 ± 0.188	2.528 ± 0.084	2.430 ± 0.115
OECA P05 L*	14.46 ± 1.93	17.01 ± 1.08	18.15 ± 0.56
OECA ΔE00 median among fruits	0.595 ± 0.051	0.793 ± 0.134	0.682 ± 0.158
OECA ΔE00 P90 among fruits	1.155 ± 0.182	1.401 ± 0.179	1.174 ± 0.246

**Table 4:** Reference colorimetry check of OECA on three cans. Values are can-level means; ΔE00 is computed between the CR-400 and OECA can means (fruits were not individually matched); SD L* summarizes between-fruit dispersion.

Can_ID	*n* Fruits	CR-400 L*/a*/b*	OECA L*/a*/b*	ΔL*	ΔE00	SD L* CR-400/OECA
C_1	34	24.40/1.20/1.34	24.18/0.65/2.25	−0.22	1.21	0.53/0.64
DGS_1	35	25.28/1.21/1.73	25.16/0.64/2.59	−0.12	1.18	0.66/1.08
DGF_5	32	24.39/1.07/1.57	25.37/0.59/2.48	+0.97	1.33	0.89/1.19

**Table 5:** ASTREE direct brine sensor responses by production batch (mean ± SD across five cans). Sensor channels are cross-sensitive responses and are not assigned one-to-one to specific tastes.

Sensor	C	DGS	DGF
AHS	2727 ± 106	3402 ± 319	2927 ± 167
PKS	303 ± 143	433 ± 129	475 ± 137
CTS	1838 ± 424	1994 ± 227	2028 ± 169
NMS	−1055 ± 61	−1030 ± 48	−1030 ± 36
CPS	151 ± 39	196 ± 28	195 ± 34
ANS	2916 ± 32	3315 ± 203	2996 ± 118
SCS	2882 ± 164	3589 ± 335	3104 ± 175

**Table 6:** Brine physicochemical variables and targeted phenolic profile by production batch.

Variable	C	DGS	DGF	Can-Level ANOVA *p*
Brine pH	7.194 ± 0.081 ^a^	8.224 ± 0.134 ^c^	7.640 ± 0.090 ^b^	<0.001
Chloride (% NaCl)	1.624 ± 0.083 ^ab^	1.656 ± 0.057 ^a^	1.522 ± 0.081 ^b^	0.036
Titratable acidity (% lactic acid)	0.031 ± 0.006	0.030 ± 0.004	0.027 ± 0.003	0.308
Hydroxytyrosol (mg/L original brine)	49.947 ± 0.916	23.387 ± 0.656	30.607 ± 0.797	—
Tyrosol (mg/L original brine)	23.453 ± 0.301	34.281 ± 1.276	29.885 ± 0.775	—
(+)-Catechin (mg/L original brine)	0.085 ± 0.088	0.901 ± 0.100	0.465 ± 0.072	—
Oleuropein (mg/L original brine)	1.524 ± 0.233	2.537 ± 0.132	3.222 ± 0.072	—
Rutin (mg/L original brine)	5.848 ± 0.087	0.044 ± 0.006	1.261 ± 0.052	—
Σ quantified phenolic compounds (mg/L original brine)	84.807 ± 1.391	62.686 ± 2.031	66.741 ± 1.714	—

Physicochemical values are mean ± SD across five cans per production batch. Overall *p* values are from one-way ANOVA and compare the three sampled production batches, with cans as sampling units within batch; they do not test the sensory conditions in general; different superscript letters within a physicochemical row indicate Tukey HSD differences at *p* < 0.05. Phenolic values are mean ± SD of three independent analytical extractions from each production batch brine sample and are expressed as mg/L of original brine as reported by the laboratory worksheet. No inferential test was applied to phenolic compounds because the production batch, rather than the analytical extraction, was the experimental unit. Σ excludes cyclohexanecarboxylic acid.

**Table 7:** Can-level PLS-DA performance using identical outer folds. Balanced accuracy (BA) is the primary metric; overall accuracy is shown together with the number of correct can predictions, while macro precision and macro F1 summarize class-wise predictive performance. Because the three classes contain the same number of cans, accuracy and BA are numerically equal in this dataset. Permutation *p* is the empirical probability of obtaining at least the observed BA after 1000 complete can label permutations. Latent variable selections by fold are reported in Table S2.

Model	Role	BA	Macro Precision	Macro F1	Accuracy (Correct/15)	Permutation *p*
S3+	Portable olfaction	0.533	0.517	0.523	0.533 (8/15)	0.050
OECA	Portable vision	0.867	0.905	0.861	0.867 (13/15)	0.001
ASTREE	Gustatory/brine block	0.933	0.944	0.933	0.933 (14/15)	0.001
S3+ + OECA	Primary portable fusion	0.600	0.587	0.583	0.600 (9/15)	0.022
S3+ + OECA + ASTREE	Expanded fusion	0.800	0.794	0.792	0.800 (12/15)	0.002
HERACLES	Commercial olfactory reference	1.000	1.000	1.000	1.000 (15/15)	0.001
IRIS	Commercial vision reference	0.867	0.878	0.866	0.867 (13/15)	0.001

HERACLES: the 13 peak area predictors were selected in AlphaSoft by class-dependent discrimination power before export, and this selection was not repeated within the outer training folds; BA = 1.000 is therefore optimistic and HERACLES should be interpreted as a reference platform result rather than as a validated classifier. Fixed hyperparameter nonlinear classifiers evaluated on the same outer folds are reported in Table S4.

**Table 8:** Instrumental sensing domains and descriptive sensory concordance.

Platform	Instrumental Domain	Sensory Attribute Considered	Relationship with Panel Assessment
S3+	Olfactory/headspace	DPP/farmyard-like off-odor	Mean LGR showed the inverse ordinal trend to DPP: LGR C > DGS > DGF, while panel DPP C < DGS < DGF.
OECA	Visual/whole fruit	Color uniformity	Between-fruit ΔE00 matched the inverse batch order of panel uniformity: ΔE00 C < DGF < DGS; panel uniformity C > DGF > DGS.
ASTREE	Gustatory/brine	Salty, bitter, acid	No direct fruit taste prediction was established; the direct brine fingerprint reflected pH and broader dissolved chemistry.
HERACLES	Olfactory/headspace	DPP/farmyard-like off-odor	Batches were completely discriminated, but no quantitative relationship with DPP intensity was established.
IRIS	Visual/whole fruit	Color/appearance	Relative color structure was consistent across batches; no quantitative relationship with panel visual scores was modeled.

Relationships are descriptive at production batch level and are not validated can-level sensory predictions, because the panel evaluated batches rather than individual cans.

**Table 9:** Summary of the chemometric models. All supervised models used the same five outer folds fixed by Can_ID (fold *k* held out C_*k*, DGS_*k*, and DGF_*k*), 1–3 latent variables chosen by inner four-fold can-level validation, autoscaling fitted on training cans, and 1000 complete can label permutations.

Data Block/Model	Method and Samples	Predictors	Preprocessing and Variable Selection	Target and Validation	Main Outcome
S3+	PCA; PLS-DA. Fifteen cans (cycles 2–4 of one jar per can averaged)	6 LGR channels	Autoscaling; no selection (pre-specified block)	C/DGS/DGF; nested can-level CV	BA 0.533 (*p* = 0.050); monotonic mean LGR gradient; DGS/DGF overlap
OECA	PCA; PLS-DA. Fifteen cans (3 trays × 34 fruits averaged)	7 (L*, a*, b*, P05 L*, SD L*, ΔE00 median and P90)	Autoscaling; pre-specified reduced block	C/DGS/DGF; nested can-level CV	BA 0.867 (*p* = 0.001); between-fruit ΔE00 ordering matched panel uniformity
ASTREE	PCA; PLS-DA. Fifteen cans (3 brine measurements averaged)	7 sensor channels	Autoscaling; no selection	C/DGS/DGF; nested can-level CV	BA 0.933 (*p* = 0.001); fingerprint covaried with brine pH between batches
HERACLES	PCA; PLS-DA. Fifteen cans (3 injections averaged)	13 peak areas	Autoscaling; peaks selected in AlphaSoft by class-dependent discrimination power before export (not nested)	C/DGS/DGF; nested can-level CV	BA 1.000 (*p* = 0.001); optimistic, reference platform
IRIS	PCA; PLS-DA. Fifteen cans (3 trays averaged)	3 (L*, a*, b*)	Autoscaling; no selection	C/DGS/DGF; nested can-level CV	BA 0.867 (*p* = 0.001); relative agreement with OECA
S3+ + OECA	Mid-level fusion PLS-DA. Fifteen cans	4 PCA scores (2 per block)	Block autoscaling and PCA fitted within training folds	C/DGS/DGF; nested can-level CV	BA 0.600 (*p* = 0.022); no gain over OECA alone
S3+ + OECA + ASTREE	Mid-level fusion PLS-DA. Fifteen cans	6 PCA scores (2 per block)	Block autoscaling and PCA fitted within training folds	C/DGS/DGF; nested can-level CV	BA 0.800 (*p* = 0.002); below best single blocks
Nonlinear check (all blocks)	RF, RBF-SVM, kNN with fixed hyperparameters	As above	Autoscaling; no tuning	Same outer folds; no permutation test	Table S4; block ranking unchanged

## Data Availability

The can-level predictor matrix with fold assignments and the analysis script are provided as Supplementary Files S1 and S2. The panel reference table, brine physicochemical and phenolic data, raw instrument exports, and the reference colorimetry workbook are available from the corresponding author on reasonable request; signed informed consent forms contain personal identifiers and are not shared.

## Supplementary Materials

The following supporting information can be downloaded at: https://www.mdpi.com/article/10.3390/s26216826/s1, Table S1: Can-level traceability across platforms (Can_ID, production batch, cross-validation fold and measurement dates of HERACLES, ASTREE, the sensory panel, S3+ and OECA/IRIS); the physical can was the independent experimental unit, fold assignments were identical across platforms, and remaining fruit was stored immersed in its own brine in hermetically closed containers at 5 °C between sessions. Table S2: PLS-DA latent variable count selected within each outer training fold; these values document model complexity and are not performance metrics. Table S3: S3+ acquisitions by can: session, order within session, mean logarithmic gas response (mean of the six channels; cycles 2–4 averaged) and chamber temperature and relative humidity recorded by the instrument during the acquisition (mean and range over cycles 2–4). Table S4: Complementary classifier check on the same five outer folds as the main PLS-DA models (balanced accuracy and correct predictions out of 15) with PLS-DA with nested latent variable selection, random forest, radial basis function support vector machine and *k*-nearest neighbors, applied to the raw variables of each block, to the within-fold PCA scores of the mid-level fusion and to low-level fusion of the raw concatenated variables; no inner tuning and no permutation test. Figure S1: Null distributions from 1000 complete can-label permutations (random seed 20261001) for the S3+ + OECA and S3+ + OECA + ASTREE fusion models; dashed lines indicate the observed balanced accuracy and p = (1 + number of permuted values ≥ observed)/1001. File S1: Can-level predictor matrix (xlsx) with Can_ID, batch, outer fold, six S3+ LGR channels, seven OECA variables, seven ASTREE channels, thirteen HERACLES peak areas and three IRIS coordinates, with a README sheet. File S2: Python script that reproduces Table 7 and Table S2, the out-of-fold class responses of Figure 7 and Figure 8, the permutation null distributions of Figure S1 and Table S4 (Python 3.11.4, NumPy 2.4.6, pandas 3.0.5, scikit-learn 1.9.0, SciPy 1.17.1; permutation seed 20261001).

## Abbreviations

The following abbreviations and instruments are used in this manuscript:

ASTREE	Potentiometric electronic tongue (Alpha MOS, Toulouse, France); sensor set #6: AHS, PKS, CTS, NMS, CPS, ANS, SCS
BA	Balanced accuracy
C, DGS, DGF	Conforming, mild farmyard-like off-odor, and strong farmyard-like off-odor production batches
ChemFET/ISFET	Chemically sensitive/ion-sensitive field-effect transistor
CIEDE2000 (ΔE00)	CIE 2000 color difference formula
CIELAB (L*, a*, b*); C*, h°	CIE 1976 color space: lightness, green-red, blue-yellow; chroma and hue angle
COI	International Olive Council
CR-400	Konica Minolta CR-400 chroma meter (reference colorimeter)
CRI	Color rendering index
CV; CVr	Cross-validation; robust coefficient of variation
D50/2°, D65/2°	CIE illuminant and 2° standard observer
DAD	Diode array detector
DPP	Predominantly perceived defect
FID	Flame ionization detector
HERACLES	Heracles Neo flash gas chromatographic electronic nose (Alpha MOS, Toulouse, France)
IQR	Interquartile range
IRIS	IRIS VA400 visual analyzer, commercial electronic eye (Alpha MOS, Toulouse, France)
kNN	*k*-nearest neighbors classifier
LED	Light-emitting diode
LGR	Logarithmic gas response, ln(*G*/*G*_0_), of one MOS channel
LV	Latent variable (PLS)
MOS	Metal–oxide semiconductor (gas sensor)
MXT-5, MXT-1701	Non-polar and mid-polar metal capillary GC columns of HERACLES
OECA (OECA ML 0.9.24)	Portable electronic eye and its software developed at CICYTEX (smartphone, LED light box, ColorChecker calibration)
P05, P90	5th and 90th percentiles
PCA; PC	Principal component analysis; principal component
PIASE	Integrated Electronic Sensory Analysis Platform (CICYTEX-INTAEX)
PLS-DA; PLS-R	Partial least squares discriminant analysis; PLS regression
RF	Random forest
S3+	Portable MOS electronic nose (Nano Sensor Systems S.r.l., NASYS, Italy); channels M1R1–M1R3, M2R1–M2R3
SD	Standard deviation
SVM (RBF)	Support vector machine with radial basis function kernel
UHPLC	Ultra-high-performance liquid chromatography

## References

[1] Sánchez R., Fernández A., Martín-Tornero E., Meléndez F., Lozano J., Martín-Vertedor D. (2022). Application of Digital Olfaction for Table Olive Industry. Sensors.

[2] Marx Í.M.G., Rodrigues N., Dias L.G., Veloso A.C.A., Pereira J.A., Drunkler D.A., Peres A.M. (2017). Sensory Classification of Table Olives Using an Electronic Tongue: Analysis of Aqueous Pastes and Brines. Talanta.

[3] Marx Í.M.G., Rodrigues N., Dias L.G., Veloso A.C.A., Pereira J.A., Drunkler D.A., Peres A.M. (2017). Assessment of Table Olives’ Organoleptic Defect Intensities Based on the Potentiometric Fingerprint Recorded by an Electronic Tongue. Food Bioprocess Technol..

[4] (2021). Method for the Sensory Analysis of Table Olives.

[5] Marx Í.M.G., Rodrigues N., Dias L.G., Veloso A.C.A., Pereira J.A., Drunkler D.A., Peres A.M. (2017). Quantification of Table Olives’ Acid, Bitter and Salty Tastes Using Potentiometric Electronic Tongue Fingerprints. LWT Food Sci. Technol..

[6] Martín-Vertedor D., Tian C., Lozano J., Monago-Maraña O., Chiappini F., Pérez-Nevado F. (2026). Discrimination of Spanish-Style Green Olives Inoculated with Undesirable Microbiota Using E-Nose, Chemometrics and Volatile Compound Profiles. Foods.

[7] Cascos G., Barea-Ramos J.D., Montero-Fernández I., Ruiz-Canales A., Lozano J., Martín-Vertedor D. (2023). Burn Defect and Phenol Prediction for Flavoured Californian-Style Black Olives Using Digital Sensors. Foods.

[8] Martín-Tornero E., Sánchez R., Lozano J., Martínez M., Arroyo P., Martín-Vertedor D. (2021). Characterization of Polyphenol and Volatile Fractions of Californian-Style Black Olives and Innovative Application of E-Nose for Acrylamide Determination. Foods.

[9] Martín-Vertedor D., Rodrigues N., Marx Í.M.G., Veloso A.C.A., Peres A.M., Pereira J.A. (2020). Impact of Thermal Sterilization on the Physicochemical-Sensory Characteristics of Californian-Style Black Olives and Its Assessment Using an Electronic Tongue. Food Control.

[10] Diaz R., Gil L., Serrano C., Blasco M., Moltó E., Blasco J. (2004). Comparison of Three Algorithms in the Classification of Table Olives by Means of Computer Vision. J. Food Eng..

[11] Riquelme M.T., Barreiro P., Ruiz-Altisent M., Valero C. (2008). Olive Classification According to External Damage Using Image Analysis. J. Food Eng..

[12] Macías-Macías M., Sánchez-Santamaria H., García Orellana C.J., González-Velasco H.M., Gallardo-Caballero R., García-Manso A. (2023). Mask R-CNN for Quality Control of Table Olives. Multimed. Tools Appl..

[13] Sharma G., Wu W., Dalal E.N. (2005). The CIEDE2000 Color-Difference Formula: Implementation Notes, Supplementary Test Data, and Mathematical Observations. Color Res. Appl..

[14] Calvini R., Pigani L. (2022). Toward the Development of Combined Artificial Sensing Systems for Food Quality Evaluation: A Review on the Application of Data Fusion of Electronic Noses, Electronic Tongues and Electronic Eyes. Sensors.

[15] Di Rosa A.R., Leone F., Cheli F., Chiofalo V. (2017). Fusion of Electronic Nose, Electronic Tongue and Computer Vision for Animal Source Food Authentication and Quality Assessment—A Review. J. Food Eng..

[16] Apetrei C., Apetrei I.M., Villanueva S., de Saja J.A., Gutierrez-Rosales F., Rodriguez-Mendez M.L. (2010). Combination of an E-Nose, an E-Tongue and an E-Eye for the Characterisation of Olive Oils with Different Degree of Bitterness. Anal. Chim. Acta.

[17] Buratti S., Malegori C., Benedetti S., Oliveri P., Giovanelli G. (2018). E-Nose, E-Tongue and E-Eye for Edible Olive Oil Characterization and Shelf Life Assessment: A Powerful Data Fusion Approach. Talanta.

[18] Johnson R.L., Mitchell A.E. (2018). Reducing Phenolics Related to Bitterness in Table Olives. J. Food Qual..

[19] Romero C., Brenes M., Yousfi K., García P., García A., Garrido A. (2004). Effect of Cultivar and Processing Method on the Contents of Polyphenols in Table Olives. J. Agric. Food Chem..

[20] García P., Romero C., García-García P., Brenes M. (2018). Bioactive Substances in Black Ripe Olives Produced in Spain and the USA. J. Food Compos. Anal..

[21] López-López A., García-García P., Durán-Quintana M.C., Garrido-Fernández A. (2004). Physicochemical and Microbiological Profile of Packed Table Olives. J. Food Prot..

[22] Garrido-Fernández A., Fernández-Díez M.J., Adams M.R. (1997). Table Olives: Production and Processing.

[23] Gobierno de España Real Decreto 679/2016, de 16 de Diciembre, por el que se Establece la Norma de Calidad de las Aceitunas de Mesa; Texto Consolidado. https://www.boe.es/eli/es/rd/2016/12/16/679/con.

[24] Wu X., Guo X., Xu X., Gao X., Xu C., Wang X., Xu B. (2026). A Combined Study Based on Electronic Tongue and Untargeted Metabolomics to Reveal Taste and Metabolite Profiles of Different Varieties Wampee (*Clausena lansium*). Food Chem. X.

[25] Hong S.J., Sung M.J., Jang G.-J., Lee S.-H. (2026). A Preliminary Study on the Characterization of Taste and Odor Attributes in Commercial Lager and Ale Beers Using E-Sensing Techniques. Front. Nutr..

[26] Calibrite. ColorChecker Classic. https://calibrite.com/us/product/colorchecker-classic/.

[27] Akkaynak D., Treibitz T., Xiao B., Gürkan U.A., Allen J.J., Demirci U., Hanlon R.T. (2014). Use of Commercial Off-the-Shelf Digital Cameras for Scientific Data Acquisition and Scene-Specific Color Calibration. J. Opt. Soc. Am. A.

[28] Wold S., Sjöström M., Eriksson L. (2001). PLS-Regression: A Basic Tool of Chemometrics. Chemom. Intell. Lab. Syst..

[29] Király P., Tóth G. (2025). Being Aware of Data Leakage and Cross-Validation Scaling in Chemometric Model Validation. J. Chemom..

[30] Pedregosa F., Varoquaux G., Gramfort A., Michel V., Thirion B., Grisel O., Blondel M., Prettenhofer P., Weiss R., Dubourg V. (2011). Scikit-Learn: Machine Learning in Python. J. Mach. Learn. Res..

[31] Virtanen P., Gommers R., Oliphant T.E., Haberland M., Reddy T., Cournapeau D., Burovski E., Peterson P., Weckesser W., Bright J. (2020). SciPy 1.0: Fundamental Algorithms for Scientific Computing in Python. Nat. Methods.

[32] Melucci D., Bendini A., Tesini F., Barbieri S., Zappi A., Vichi S., Conte L., Gallina Toschi T. (2016). Rapid Direct Analysis to Discriminate Geographic Origin of Extra Virgin Olive Oils by Flash Gas Chromatography Electronic Nose and Chemometrics. Food Chem..

[33] Barbieri S., Cevoli C., Bendini A., Quintanilla-Casas B., García-González D.L., Gallina Toschi T. (2020). Flash Gas Chromatography in Tandem with Chemometrics: A Rapid Screening Tool for Quality Grades of Virgin Olive Oils. Foods.

[34] Cai W., Zhou W., Liu J., Wang J., Kuang D., Wang J., Long Q., Huang D. (2024). An Exploratory Study on the Rapid Detection of Volatile Organic Compounds in Gardenia Fruit Using the Heracles NEO Ultra-Fast Gas Phase Electronic Nose. Metabolites.

