# Supplementary material for: Toward the Digitalization of Sensory Analysis of Black Table Olives Using Electronic Nose, Tongue, and Eye
Source: Sensors (Basel). 2026 Oct 28;26(21):6826. doi: 10.3390/s26216826 (PMC13765641; doi:10.3390/s26216826)
Supplement: Supplementary file 1 [file sensors-26-06826-s001.zip › sensors-4638503-supplementary.pdf]

Supplementary Materials

Toward the Digitalization of Sensory Analysis of Black Table Olives Using Electronic Nose, Tongue, and Eye

Ramiro Sánchez Baltasar

Table S1. Can-level traceability across platforms.

The physical can was the independent experimental unit. Fold assignments were identical across instrumental platforms. Remaining fruit was stored immersed in its own brine in hermetically closed containers at 5 °C between analytical sessions.

Can_ID	Batch	CV fold	HERACLES	ASTREE	Sensory panel	S3+	OECA / IRIS
C_1	C	1	2026-03-20	2026-04-06	2026-07-29	2026-09-18	2026-09-30
C_2	C	2	2026-03-20	2026-04-06	2026-07-29	2026-09-15	2026-09-30
C_3	C	3	2026-03-20	2026-04-06	2026-07-29	2026-09-16	2026-09-30
C_4	C	4	2026-03-20	2026-04-06	2026-07-29	2026-09-17	2026-09-30
C_5	C	5	2026-03-20	2026-04-06	2026-07-29	2026-09-18	2026-09-30
DGS_1	DGS	1	2026-03-20	2026-04-06	2026-07-29	2026-09-15	2026-09-30
DGS_2	DGS	2	2026-03-20	2026-04-06	2026-07-29	2026-09-15	2026-09-30
DGS_3	DGS	3	2026-03-20	2026-04-06	2026-07-29	2026-09-16	2026-09-30
DGS_4	DGS	4	2026-03-20	2026-04-06	2026-07-29	2026-09-17	2026-09-30
DGS_5	DGS	5	2026-03-20	2026-04-06	2026-07-29	2026-09-18	2026-09-30
DGF_1	DGF	1	2026-03-20	2026-04-06	2026-07-29	2026-09-15	2026-09-30
DGF_2	DGF	2	2026-03-20	2026-04-06	2026-07-29	2026-09-15	2026-09-30
DGF_3	DGF	3	2026-03-20	2026-04-06	2026-07-29	2026-09-16	2026-09-30
DGF_4	DGF	4	2026-03-20	2026-04-06	2026-07-29	2026-09-17	2026-09-30
DGF_5	DGF	5	2026-03-20	2026-04-06	2026-07-29	2026-09-18	2026-09-30

Note: sensory assessment was performed at production batch level on freshly opened cans of the same batches (not on the 15 matched cans); the date is repeated only to document temporal correspondence with the physical cans. S3+ dates correspond to the retained acquisitions used in the analysis.

Table S2. PLS-DA latent variable count selected within each outer training fold. These values document model complexity; they are not performance metrics.

Model	Fold 1	Fold 2	Fold 3	Fold 4	Fold 5
S3+	1	1	2	1	2
OECA	3	3	2	3	3
ASTREE	2	3	3	3	3
S3+ + OECA	2	2	1	2	2
S3+ + OECA + ASTREE	2	1	2	2	2
HERACLES	2	2	2	2	2
IRIS	2	3	2	2	2

Table S3. S3+ acquisitions by can: session, order within session, mean logarithmic gas response (LGR, mean of the six channels; cycles 2–4 averaged) and chamber temperature and relative humidity recorded by the instrument during the acquisition (mean and range over cycles 2–4). Two cans (DGS_1 and DGF_4) were acquired in a higher chamber temperature regime and DGS_4 transitioned between regimes; excluding DGS_1 and DGF_4 left the batch ordering of mean LGR unchanged (C 0.871, DGS 0.765, DGF 0.683).

Can_ID	Batch	Session/order	Mean LGR	Chamber T (°C)	Chamber RH (%)
DGS_1	DGS	2026-09-15 #1	0.666	65.1 (64.1–66.0)	9.5 (8.9–11.3)
DGF_1	DGF	2026-09-15 #2	0.599	48.7 (47.9–49.6)	15.1 (13.8–18.6)
DGF_2	DGF	2026-09-15 #3	0.624	48.9 (48.2–49.3)	14.7 (13.8–18.3)
C_2	C	2026-09-15 #4	0.865	48.3 (47.0–48.7)	14.8 (13.9–18.4)

DGS_2	DGS	2026-09-15 #5	0.677	48.3 (47.3–48.8)	15.2 (14.1–19.0)
DGS_3	DGS	2026-09-16 #1	0.793	47.8 (47.4–48.1)	17.1 (16.4–19.7)
C_3	C	2026-09-16 #2	0.834	48.4 (47.8–48.9)	17.0 (16.2–20.2)
DGF_3	DGF	2026-09-16 #3	0.729	49.2 (48.1–49.7)	16.6 (15.7–20.2)
DGF_4	DGF	2026-09-17 #1	0.647	63.5 (61.6–64.4)	9.3 (8.9–11.2)
DGS_4	DGS	2026-09-17 #2	0.709	52.8 (47.3–63.4)	12.8 (9.1–17.7)
C_4	C	2026-09-17 #3	0.833	47.1 (45.4–48.0)	15.4 (14.3–19.0)
C_5	C	2026-09-18 #1	0.954	48.1 (47.6–48.5)	14.3 (13.4–17.6)
DGS_5	DGS	2026-09-18 #2	0.882	48.9 (48.2–49.4)	13.9 (13.0–17.4)
DGF_5	DGF	2026-09-18 #3	0.781	49.0 (48.6–49.7)	14.3 (13.4–17.8)
C_1	C	2026-09-18 #4	0.868	48.4 (47.0–49.1)	14.8 (13.8–18.4)

Table S4. Complementary classifier check on the same five outer folds as the main PLS-DA models (balanced accuracy, BA; correct predictions out of 15). Inputs: the raw autoscaled variables of each single block; for the two fused models, the within-fold PCA scores of the pre-specified mid-level fusion (as in the main text) and, in addition, low-level fusion, i.e., the raw variables of all blocks concatenated after block-wise autoscaling. Learners: PLS-DA with nested latent variable selection (reference; identical to Table 7 for the main models), random forest (RF, 500 trees, scikit-learn defaults otherwise), radial basis function support vector machine (SVM-RBF, C = 1, gamma = 'scale') and k-nearest neighbors (kNN, k = 3), all trained on training fold data without inner tuning, which 15 cans do not support; no permutation test was run for this check. Low-level fusion reached BA 0.733 (S3+ + OECA) with every learner and at most 0.867 (S3+ + OECA + ASTREE), equal to OECA alone and below ASTREE (0.933) and HERACLES (1.000); no learner exceeded the best single-block PLS-DA results, and the mid-level fusion models remained at or below BA 0.800.

Inputs/model	PLS-DA (nested LV)	RF	SVM-RBF	kNN
S3+	0.533 (8/15)	0.600 (9/15)	0.533 (8/15)	0.667 (10/15)
OECA	0.867 (13/15)	0.867 (13/15)	0.667 (10/15)	0.733 (11/15)
ASTREE	0.933 (14/15)	0.800 (12/15)	0.800 (12/15)	0.733 (11/15)
HERACLES	1.000 (15/15)	1.000 (15/15)	1.000 (15/15)	1.000 (15/15)
IRIS	0.867 (13/15)	0.800 (12/15)	0.933 (14/15)	0.867 (13/15)
S3+ + OECA (mid-level, PCA scores; main text)	0.600 (9/15)	0.600 (9/15)	0.667 (10/15)	0.600 (9/15)
S3+ + OECA + ASTREE (mid-level, PCA scores; main text)	0.800 (12/15)	0.733 (11/15)	0.800 (12/15)	0.667 (10/15)
S3+ + OECA (low-level, raw variables)	0.733 (11/15)	0.733 (11/15)	0.733 (11/15)	0.733 (11/15)
S3+ + OECA + ASTREE (low-level, raw variables)	0.867 (13/15)	0.867 (13/15)	0.800 (12/15)	0.867 (13/15)

Figure S1. Permutation test distributions for the fusion models.

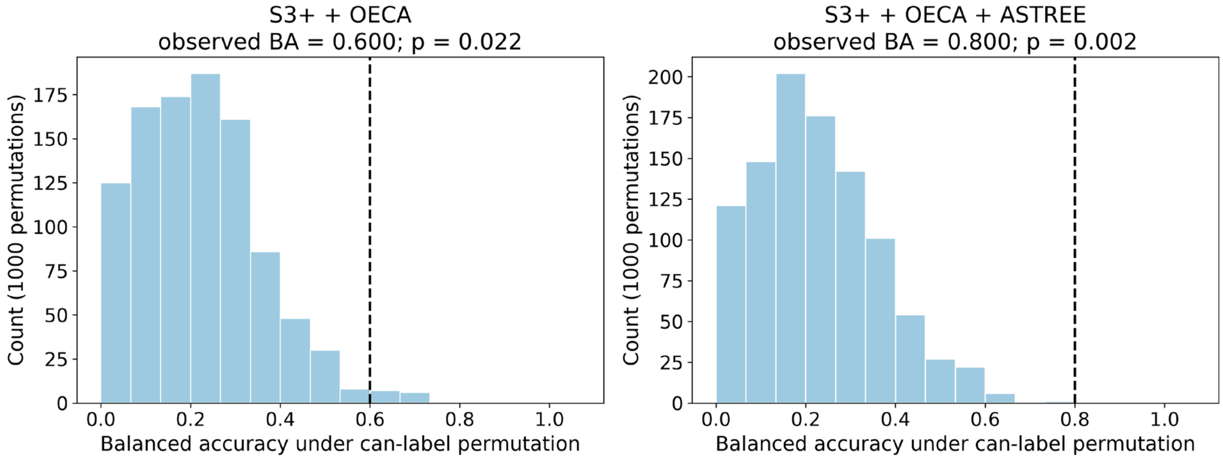

Figure S1. Null distributions from 1000 complete can label permutations (random seed 20261001) for the S3+ + OECA portable fusion and the S3+ + OECA + ASTREE expanded fusion, regenerated with File S2. Dashed lines indicate the observed balanced accuracy; p = (1 + number of permuted values ≥ observed)/1001.

Files S1 and S2. Can-level predictor matrix and analysis code

File S1 (File_S1_can_level_predictor_matrix.xlsx) contains the 15 × 36 can-level predictor matrix used for all PCA, PLS-DA, fusion and permutation analyses: Can_ID, batch, outer fold, six S3+ LGR channels, seven OECA variables, seven ASTREE channels, thirteen HERACLES peak areas and three IRIS coordinates, with a README sheet describing each block.

File S2 (File_S2_multisensor_plsda.py) is the Python script that reproduces Table 7 (balanced accuracy, macro precision, macro F1, accuracy and permutation p values), Table S2 (latent variables per outer fold), the out-of-fold class responses plotted in Figures 7 and 8, the permutation null distributions of Figure S1 and the classifier check of Table S4, including the low-level fusion inputs. Software versions used: Python 3.11.4, numpy 2.4.6, pandas 3.0.5, scikit-learn 1.9.0 and SciPy 1.17.1; permutation seed 20261001 (numpy default_rng). Usage: python File_S2_multisensor_plsda.py File_S1_can_level_predictor_matrix.xlsx OUTPUT_DIR.
